# Joint learning improves protein abundance prediction in cancers

**DOI:** 10.1186/s12915-019-0730-9

**Published:** 2019-12-23

**Authors:** Hongyang Li, Omer Siddiqui, Hongjiu Zhang, Yuanfang Guan

**Affiliations:** 1 Department of Computational Medicine and Bioinformatics, University of Michigan, 100 Washtenaw Avenue, Ann Arbor, MI 48109 USA; 2 Department of Internal Medicine, University of Michigan, 100 Washtenaw Avenue, Ann Arbor, MI 48109 USA

**Keywords:** Cancer, Proteomics, Transcriptomics, Machine learning

## Abstract

**Background:**

The classic central dogma in biology is the information flow from DNA to mRNA to protein, yet complicated regulatory mechanisms underlying protein translation often lead to weak correlations between mRNA and protein abundances. This is particularly the case in cancer samples and when evaluating the same gene across multiple samples.

**Results:**

Here, we report a method for predicting proteome from transcriptome, using a training dataset provided by NCI-CPTAC and TCGA, consisting of transcriptome and proteome data from 77 breast and 105 ovarian cancer samples. First, we establish a generic model capturing the correlation between mRNA and protein abundance of a single gene. Second, we build a gene-specific model capturing the interdependencies among multiple genes in a regulatory network. Third, we create a cross-tissue model by joint learning the information of shared regulatory networks and pathways across cancer tissues. Our method ranked first in the NCI-CPTAC DREAM Proteogenomics Challenge, and the predictive performance is close to the accuracy of experimental replicates. Key functional pathways and network modules controlling the proteomic abundance in cancers were revealed, in particular metabolism-related genes.

**Conclusions:**

We present a method to predict proteome from transcriptome, leveraging data from different cancer tissues to build a trans-tissue model, and suggest how to integrate information from multiple cancers to provide a foundation for further research.

## Background

The central dogma of information flow from DNA to mRNA to protein has been applied for nearly six decades [1]. Yet, the cell functions as a whole: besides the translation from mRNA to protein, many other features are important to the complex protein expression process, including microRNA, upstream open reading frame [2], cap-binding proteins [3], poly(A) tails [4], nonsense-mediated decay [5], or alternative splicing [6]. In addition, the mRNA and protein abundances are dynamic, due to ubiquitination and other degradation mechanisms to fulfill diverse condition-dependent functional requirements [7]. These complicated regulatory mechanisms underlying protein translation lead to the weak correlations between mRNA and protein abundances, when evaluating the same gene across multiple samples [7–12]. Identifying the missing factors affecting transcriptomic and proteomic correlation is important to understanding the biological mechanisms behind phenotypic variances and diseases.

This is particularly true in cancers. Transcriptomic and proteomic variations across individuals are expected in diverse cancers, such as colorectal, breast, and ovarian cancers [10–12]. These variations have important clinical consequences and implications, due to activation of different functional pathways, leading to different subtypes in the same organ, and biomarkers indicative of high- and low-risk patients in survival analysis [10–12]. These transcriptional and proteomic expression profiles provide invaluable information to studying cancer mechanisms. However, compared with the fast, inexpensive RNA sequencing profiles, large-scale high-quality proteomic data are costly to obtain, despite remarkable progress. Therefore, a computational model to predict protein abundance from mRNA data could help not only to quickly obtain an estimation of proteomic data, but also to understand what are the important players in cancers.

The National Cancer Institute (NCI) Clinical Proteomic Tumor Analysis Consortium (CPTAC) [13] and The Cancer Genome Atlas (TCGA) provide large datasets of proteomic and transcriptomic data in many cancers, which is an unprecedented source for exploring the regulatory process of protein expression. In 2017, the Dialogue on Reverse Engineering Assessment and Method (DREAM) [14] organized the NCI-CPTAC Proteogenomics Challenge. This challenge provides a systematic benchmark to evaluate computational methods for predicting proteomic profiles in breast and ovarian cancers. Here, we describe the best-performing algorithm in this challenge and reveal the insights derived. Our approach pinpoints the relative importance of the innate correlations between mRNA and protein levels, and the global direct and indirect interactions across all genes in controlling the expression level of a protein. Based on the intuition that the regulatory mechanism may be shared across different cancer types, we built a new model that shares parameters across two cancers, and improved prediction performance in both cancers. This reveals a new, unexplored aspect of the regulatory mechanism that is previously not captured in single tissue modeling approaches. Pathway analysis and gene-gene interaction network indicate that functionally different gene sets have different predictability profiles and regulatory powers. In sum, our approach offers a new field standard for protein abundance prediction across cancer patients, and the key features used in our model and the innovation of joint learning across two cancer types will be instructive for future method development and protein expression regulatory mechanism exploration.

## Results

### Overview of the experimental design for protein abundance prediction

**Fig. 1:**
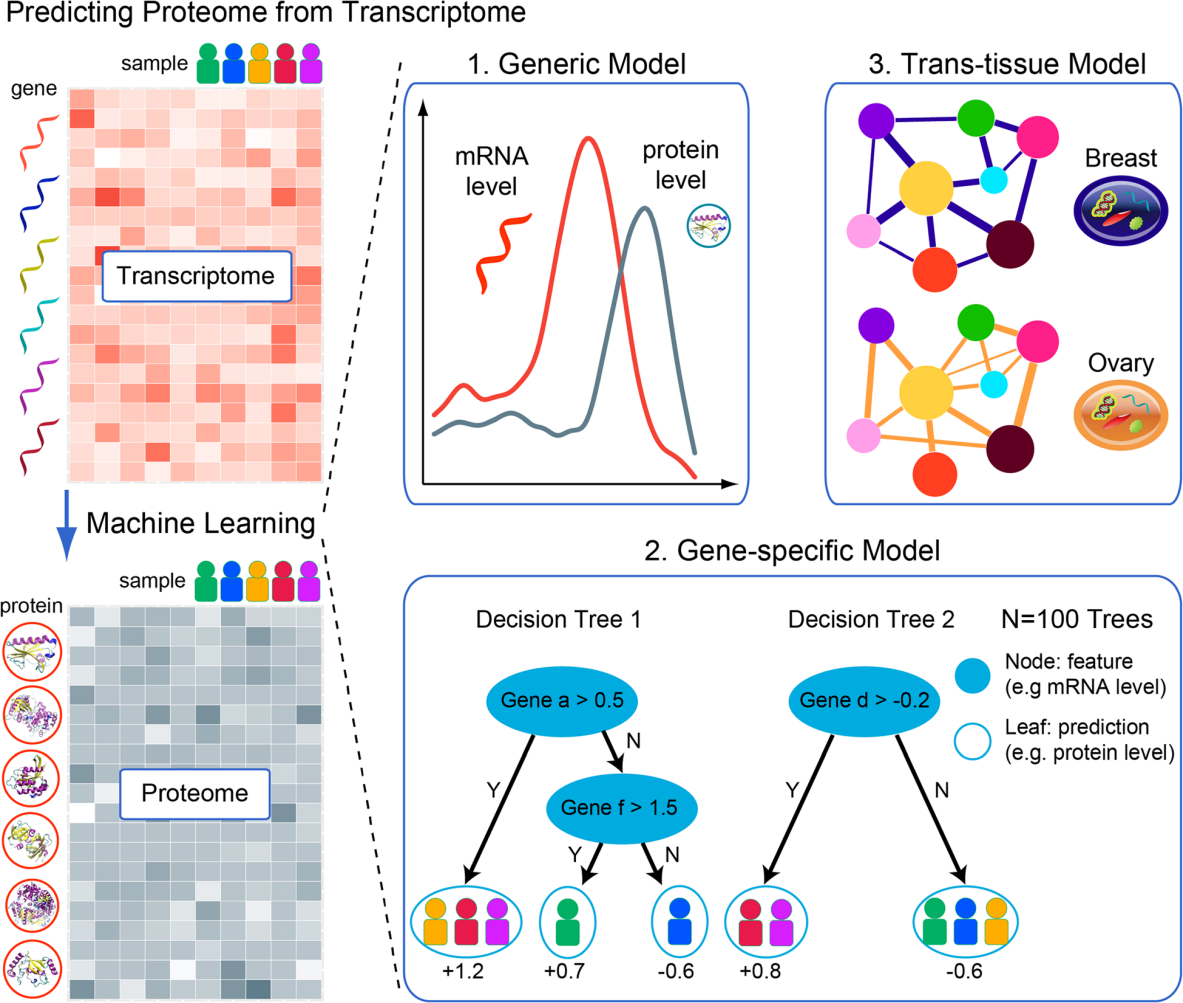
Overview of the algorithm design for predicting proteomic expression from transcriptomic data. The overall task of this study is to transform the red matrix, representing the transcriptomic level expression across different individuals, to the blue-gray matrix, representing the proteomic level expression (left). Three models are created to address this problem (right): (1) generic model, which captures the innate correlation between mRNA and protein level; (2) gene-specific model, which captures how multiple genes work in a network to control the protein level under investigation through random forest aggregation of multiple base learners; and (3) trans-tissue model, which captures the shared functional networks across cancer types

In this study, we use a training dataset provided by NCI-CPTAC, which consists of the transcriptome and proteome data from 77 breast and 105 ovarian cancer samples. To unbiasedly evaluate prediction methods, a docker image system was used in the NCI-CPTAC DREAM challenge for participants to submit their code and score on a held-out testing dataset of proteomic data from 108 breast and 82 ovarian cancer samples (Fig. 1 left). For each protein, the primary evaluation metric was Pearson’s correlation between predictions and observations across samples. The final score was calculated by averaging the prediction correlations of all proteins under consideration. In addition, the normalized root mean square error (NRMSE) between predictions and observations was used as the secondary scoring metrics to evaluate models.

We developed three major components in order to extract informative features and exploit the training data (Fig. 1 right). First, the intrinsic correlation between mRNA and protein levels was considered in the generic model. Second, for each protein under investigation, we utilized the nonlinear interdependencies among all genes in the gene-specific model. Third, the model weights were interchangeable between cancer tissues, capturing the shared regulatory mechanism in the trans-tissue model. By integrating these components, we enhanced the prediction of protein abundance in both breast and ovarian cancers.

### Dissection of critical components in determining protein abundance

**Fig. 2:**
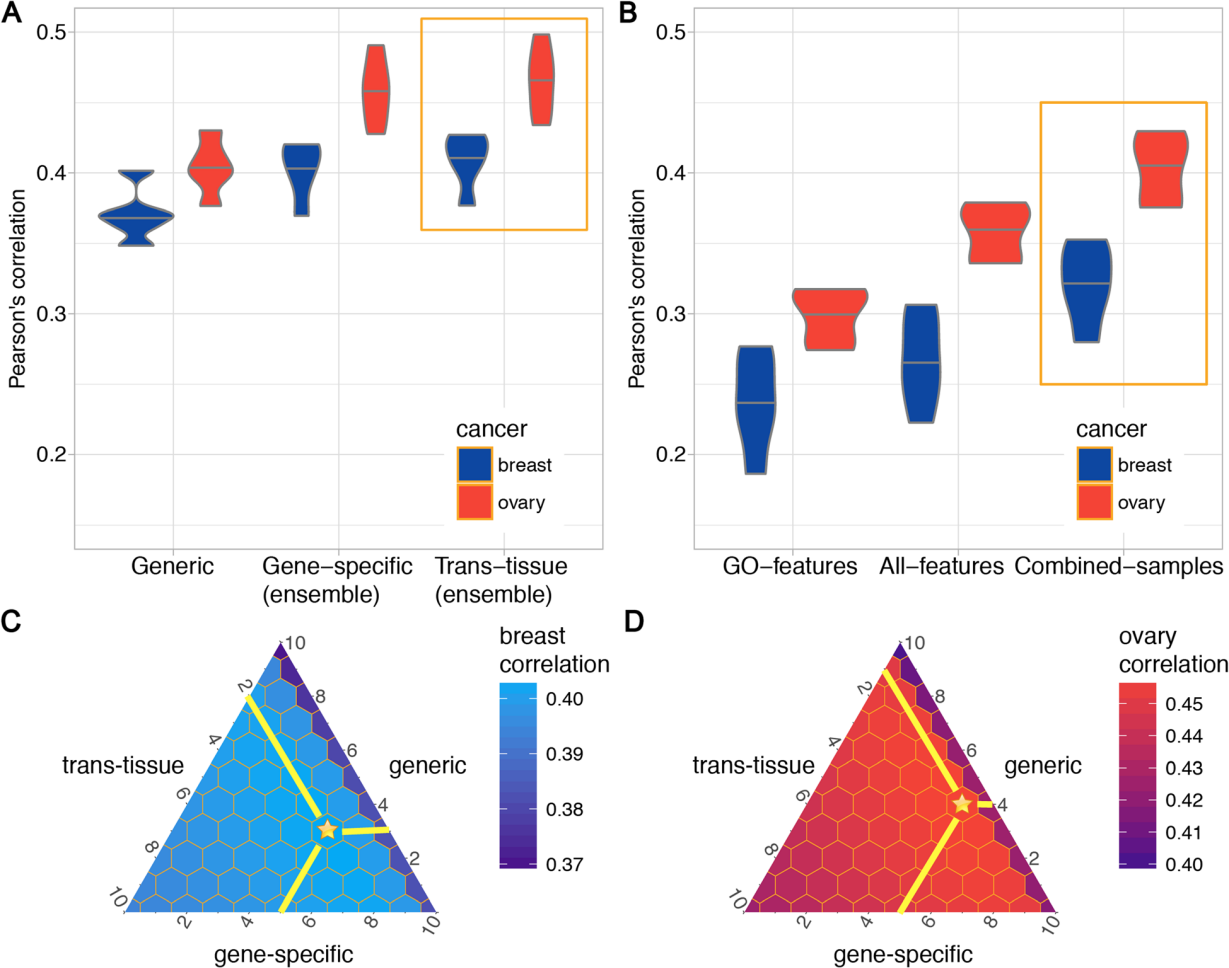
The contributions of different models to proteome prediction in breast and ovarian cancers. **a** From left to right, the correlations were calculated by assembling the following three models step by step (blue: breast; red: ovary): (1) The generic model, which only uses the transcript-level expression of a target protein as the only feature; (2) the gene-specific model, which uses the transcript-level expressions of all genes as features for predicting a target protein; and (3) the trans-tissue model, which is similar to the gene-specific model yet combines both breast and ovarian cancer samples. **b** Dissection of the gene-specific model by using different sets of features and samples. (1) Sub-selecting all genes related to “gene expression” as features. (2) Using all transcripts as features to predict the target protein. (3) Combining samples from two tissues to train. The correlations between all pairs of models are significantly different (*p* < 2.2e−16) using Wilcoxon signed-rank test, after bootstrap sampling for 1000 times. **c**, **d.** The contributions of the generic, gene-specific, and trans-tissue models to the final predictions in the **c** breast and **d** ovary. Each grid within a triangle represents the combination of three models, and the distances to three edges correspond to three weights. If a grid is far away from an edge, it means the corresponding model has a large weight. The combination that achieves the highest correlation is labeled by the golden star, where the best combination weights of the generic, gene-specific, and trans-tissue models are 2:3:5 in the breast and 1:4:5 in the ovary. Notably, the right arms of both triangles are in “darker” color (lower correlations), representing large correlation increases when the generic model are integrated

To quantify the relative contributions of features that determine protein abundance, we investigated the performance gain of each component. The average Pearson’s correlations of the generic model were 0.37 and 0.40 in breast and ovarian cancer, respectively (Fig. 2a left; Additional file 1: Figure S1–S2; Additional file 2: Table S1). By combining the predictions from the generic and the gene-specific models, we significantly improved the correlations to 0.40 (breast) and 0.46 (ovary) (Fig. 2a middle; *p* < 2.2e−16; see the “Methods” section). To consider the similarity across cancer tissues, we further integrated the predictions from the trans-tissue model and achieved the highest correlations of 0.41 (breast) and 0.47 (ovary) (Fig. 2a right; *p* < 2.2e−16; see the “Methods” section). In addition, the RMSEs of these components were also calculated (Additional file 1: Figure S3A and Figure S4–S5).

When we built the gene-specific model, a key question was how many genes should be used as features for predicting protein abundance. As we expected, as the number of features increased (the top 10, 100, or 1000 expressed genes), the predictive performances consistently improved in terms of both correlation (Additional file 1: Figure S6–S8; Additional file 2: Table S2) and RMSE (Additional file 1: Figure S9–S11). Interestingly, filtering feature genes based on prior knowledge of Gene Ontology (GO) [15, 16] related to “translation” and “gene expression” did not improve the performance, whereas using all genes as features achieved the highest correlations (“GO-features” and “All-features” in Fig. 2b; Additional file 1: Figure S12–S13) and lowest RMSEs (Additional file 1: Figure S3B and Figure S14–S15). Of note, the violins in Fig. 2a represent the ensemble predictions from (i) generic, (ii) generic and gene-specific, and (iii) generic, gene-specific, and trans-tissue models, whereas the violins in Fig. 2b represent the gene-specific model without stacking any other models. As a result, the correlations in Fig. 2a are overall higher than those in Fig. 2b. These results indicate that the abundance of a single protein is regulated by the commonly existing gene-gene associations; the regulatory contributions are not from a small set of genes but universally distributed among all genes.

To further investigate the contributions of these three models, we performed the grid-search of various weights of them (Additional file 2: Table S3–S4). To be specific, we used w1, w2, and w3 to denote the weights for predictions from the (i) generic, (ii) gene-specific, and (iii) trans-tissue models, respectively. The sum of these three weights was set to a constant of 10 and all possible combinations of non-negative integers were tested, resulting in the grid-search triangles of three models in Fig. 2c, d. In each triangle, the three edges represent three models and the numbers along each edge represent the stacking weights. Each grid within a triangle represents the combination of three models and the distances to three edges correspond to three weights. If a grid is far away from an edge, it means the corresponding model has a large weight. For example, in Fig. 2c, the golden star is 2 grids away from the right edge (yellow horizontal line), corresponding to the weight of 2 for the generic model. Similarly, we can calculate the distances from the golden star to the other two edges and obtain the corresponding weights of 3 and 5. The golden star therefore represents the combination weights of 2:3:5 = generic:gene-specific:trans-tissue. We observed similar “dark” right arms of the ternary plots in both breast and ovarian cancers (Fig. 2c, d), where the correlations were relatively low. This is because the gene-specific and trans-tissue models captured non-redundant regulatory information, compared with the generic model. When integrating different types of models, we significantly improved the correlations, leading to the sudden color change moving from the right arms towards the left-bottom. Furthermore, when moving along the right arms towards the bottom right, the correlation gradually increased (the color becomes brighter), since the trans-tissue model contributed more to the final prediction. The best combination weights of the generic, gene-specific, and trans-tissue models were 2:3:5 in breast and 1:4:5 in the ovary, where the trans-tissue model had the largest weights in both cancers (golden stars in Fig. 2c, d).

### Regulatory information of protein abundance is transferable between breast and ovarian cancers

Regulatory pathways are expected to be shared to a certain extent across different tissues, which motivates us to develop a model that shares the weights between tissues. To investigate the effect of transferring information between cancer tissues, we trained a “Combined-samples” model by combining samples from these two cancers and directly compared it with the model training on one cancer only. The “Combined-samples” model largely increased the prediction correlation from 0.27 to 0.32 in the breast and from 0.36 to 0.49 in the ovary (“All-features” and “Combined-samples” in Fig. 2b). In fact, the performance was highly dependent on the number of training samples. When we used 40%, 60%, 80%, or 100% of the samples to train the model, the performances gradually increased in terms of both correlation (Additional file 1: Figure S16–S18; Additional file 2: Table S5) and RMSE (Additional file 1: Figure S19–S21). These results demonstrate that current prediction performance is limited by the relatively small sample size. Therefore, we combined samples from the two types of cancers and trained the trans-tissue model, assuming that the same protein is regulated in a similar fashion in these two cancers. As we expected, the trans-tissue model achieved higher correlations since it was trained on more samples.

**Fig. 3:**
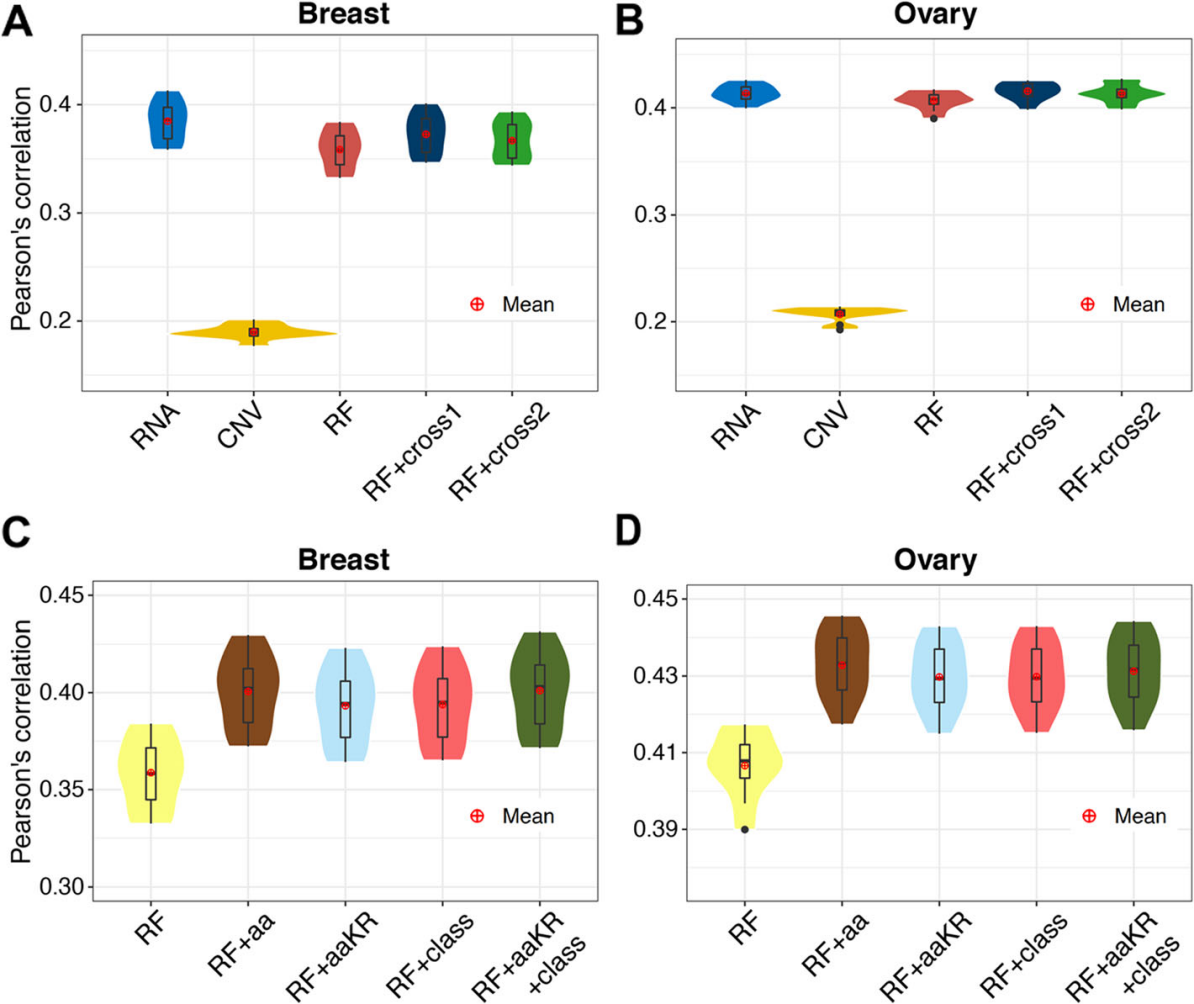
Prediction performance using different input features. **a**, **b** Pearson’s correlation between predictions and observations across patients in the **a** breast and **b** ovary. The *x*-axis represents different methods. Specifically, RNA and CNV simply use the mRNA and DNA copy number variation values as approximations for the proteomic values, respectively. RF is the random forest model trained across all available proteins using two features, the corresponding RNA and CNV values of a protein. RF+cross1 and RF+cross2 are the random forest models transferring information cross breast and ovarian cancers. In RF+cross1, we trained two RF models on breast or ovary data separately and assembled the predictions of them, while in RF+cross2, we only trained one RF model on the combined breast and ovary data. **c**, **d**. The prediction performance using protein sequence and class information in the (**c**) breast and (**d**) ovary. In addition to RNA and CNV, in RF+aa, we add 20 features, each representing the number of occurrence of an amino acid in a protein. In RF+aaKR, we add only the numbers of two amino acids, lysine (K) and arginine (R), which are the cleavage targets of trypsin in proteomics mass spectrometry. In RF+class, we add four binary features, representing the four protein classes defined by the CATH protein structure classification database. In RF+aaKR+class, we add features of both the number of amino acids and protein classes

In addition to the transcriptomic data, we also investigated other types of data that could potentially contribute to the prediction of protein abundance (Fig. 3). We first considered DNA copy number variation (CNV) as the approximation for proteome. Compared with RNA, CNV provided much less information and the prediction correlation of CNV itself was only 0.2 in both breast and ovarian cancers (RNA and CNV in Fig. 3a, b). We next used the RNA and CNV values of a gene as features and trained a random forest model on all available proteins, yet the performance was worse than RNA itself. Nevertheless, the cross-tissue models either trained on separated or combined data improves the correlation (“RF,” “RF+cross1,” and “RF+cross2” in Fig. 3a, b). These results indicate that the RNA level itself is already a good approximation for the protein abundance, better than CNV or the simple model trained on RNA and CNV. Therefore, the CNV data was not used in our final model. To reduce the potential batch effects across individuals, different normalization methods were also tested (Additional file 1: Figure S22).

We further explored the effects of adding features of protein sequence and class. For each amino acid, we counted the number of occurrence in a protein sequence as an extra feature, improving the correlations in both cancers (“RF+aa” and “RF+aaKR” in Fig. 3c, d). The biological motivation of engineering these amino acid features is that the expressions of different proteins are assumed to be regulated by different functional pathways. These differences among different proteins should be integrated into machine learning models, and the amino acid composition is a simple and effective way to encode this information. In addition, we focus on two amino acids, lysine (K) and arginine (R), which are the cleavage targets of trypsin in proteomics mass spectrometry. Similarly, we considered the protein classes defined by CATH protein structure classification database as extra features, which also improved the performance (“RF+class” and “RF+aaKR+class” in Fig. 3c, d). These results indicate that biological knowledge, including amino acid composition and protein classes, is helpful in predicting protein abundance. In fact, a similar approach of amino acid composition has been used previously in predicting protein crystallization [17]. When assembling models using these features into the final model, we did not observe any improvement. These features were therefore not used in our final model.

### Joint learning approaches experimental replicate level accuracy

Since proteomics data have intrinsic noises due to batch effects and fluctuations, we further estimated the theoretical best performance based on the experimental replicates for the overlapping samples measured at two different cohorts. To be specific, there are 32 ovarian cancer samples measured at both JHU and PNNL. For these samples, we calculated Pearson’s correlation (0.59) and RMSE (0.179) between the experimental replicates at two cohorts (Additional file 2: Table S6). Meanwhile, the prediction correlation and RMSE of our method on the held-out testing dataset during the NCI-CPTAC DREAM challenge (Additional file 2: Table S7) were 0.53 (Additional file 1: Figure S23–S24) and 0.186 (Additional file 1: Figure S25–S26), respectively. These results indicate that the protein abundance prediction is a relatively hard task, due to the intrinsic noises of the measurements across cancer samples. Although our method only achieved a medium prediction correlation of 0.53, it is in fact close to the correlation of 0.59 between experimental replicates. In terms of RMSE, our method is even closer to the accuracy of experimental replicates and the error is only 3.9% higher, which is calculated from (0.186 − 0.179)/0.179 = 3.9%. Currently, our method was built on 77 breast and 105 ovarian cancer samples by transfer learning. We foresee that this method would become even closer to the performance of experimental replicates with more training samples, since we have observed the gradually increased performance as the training set becomes larger (Additional file 1: Figure S16).

### Functionally diverse gene sets display different predictability spectrums

**Fig. 4:**
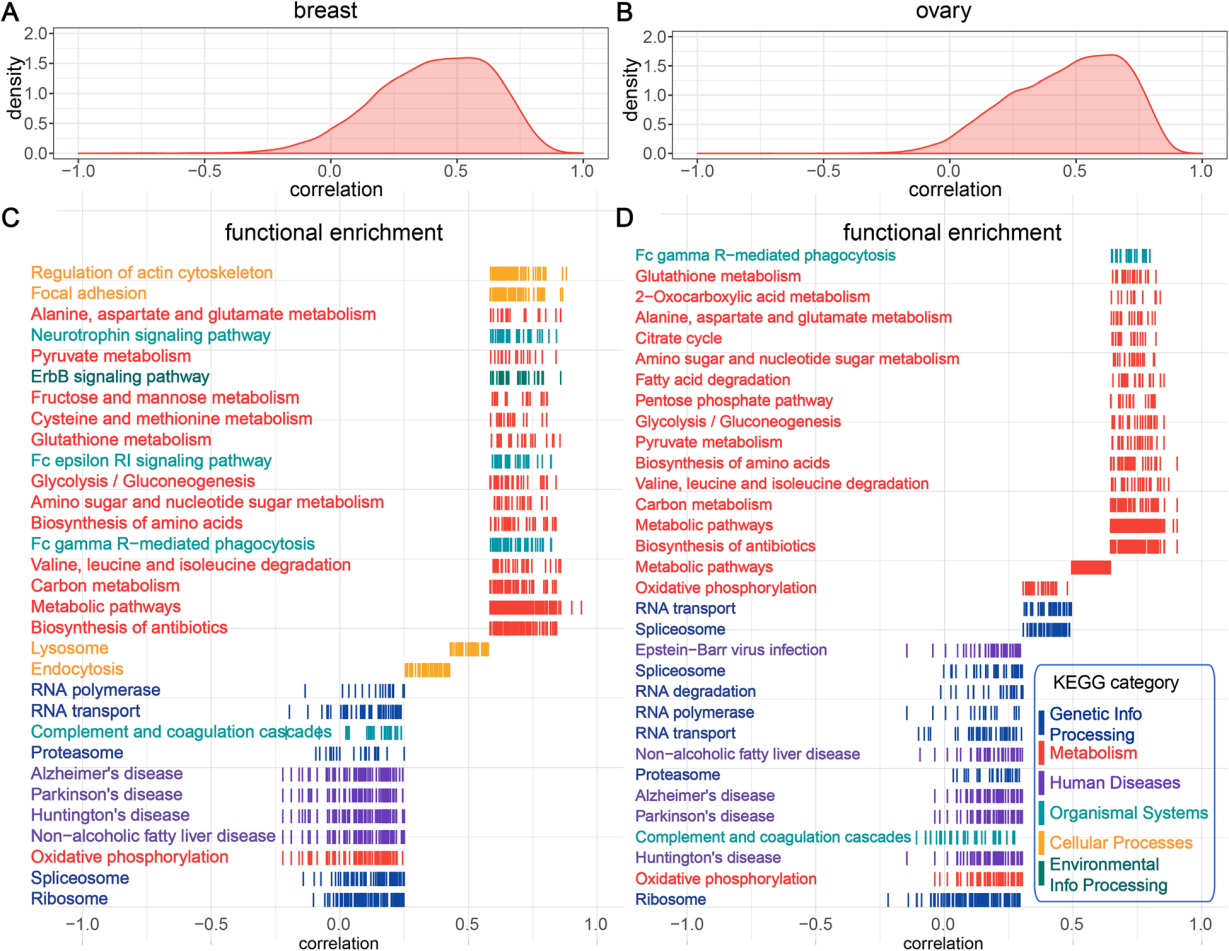
The functional enrichment analysis of gene sets with different predictability spectrums. **a**, **b** The overall distribution of Pearson’s correlations between observations and our predictions in **a** breast and **b** ovarian cancers. **c**, **d** Functional enrichment analysis was performed on gene subsets based on the predictability. The colors represent the major KEGG categories. Genes with high prediction correlations are mainly associated with “Metabolism,” whereas genes with low prediction correlations are mainly associated with “Genetic Information Processing” and “Human Diseases”

To investigate the relationship between protein functions and ease of predictability, we performed functional enrichment analysis of all considered proteins. We found that gene sets of different predictability were functionally enriched in different Kyoto Encyclopedia of Genes and Genomes (KEGG) pathways [18]. The overall distributions of correlations between our predictions and observations for breast and ovarian cancers are shown in Fig. 4 a and b, respectively. Based on the predictability, we partitioned the proteins into four groups: the top 0–25% easiest proteins to predict, the median 25–50% and 50–75% predictable group, and the bottom 75–100% hardest proteins to predict. For each group, the functional enrichment analysis was performed against KEGG pathways. In the breast cancer, the gene group easy to predict was highly associated with the “Metabolism” category, including pathways of amino acids and other biomolecules metabolism (red genes in Fig. 4c). In contrast, the genes hard to predict were usually associated with the “Genetic Information Processing” and “Human Disease” categories, including pathways of ribosome, spliceosome, proteasome, and three neurodegenerative diseases (blue and purple genes in Fig. 4c, respectively). Interestingly, it has been reported that cancers and neurodegenerative disease share common mechanisms of molecular abnormalities [19, 20]. In particular, microRNA (miRNA)-based regulation of mRNA translation is a potential common regulator of both cancer and neurodegenerative disease [21]. Mutations in genes associated with cell cycle regulation, protein turnover, and DNA repair have been implicated in these two types of diseases [22]. We observed similar distribution of functionally different gene sets in ovarian cancers (Fig. 4d). These results are consistent with the previous observations that stable and housekeeping proteins usually have weak mRNA-protein correlations, whereas dynamic proteins tend to have strong correlations [10–12].

To further understand the regulatory patterns of different genes, we performed similar functional enrichment analysis on genes ranked by the prediction improvement after integrating the gene-gene interdependencies of the gene-specific model. We found that in general the housekeeping proteins, associated with RNA transport, ribosome, spliceosome, and proteasome, benefited more than the metabolism-related genes in both cancers (Additional file 1: Figure S27). In addition, several disease-related gene sets gained relatively large improvements in the ovarian cancer, including Parkinson’s, Alzheimer’s, and Huntington’s diseases. In sum, we find similar mapping landscapes between protein abundance prediction improvement and functional pathways in breast and ovarian cancers.

### Metabolism-related genes are essential in regulating the protein abundance

**Fig. 5:**
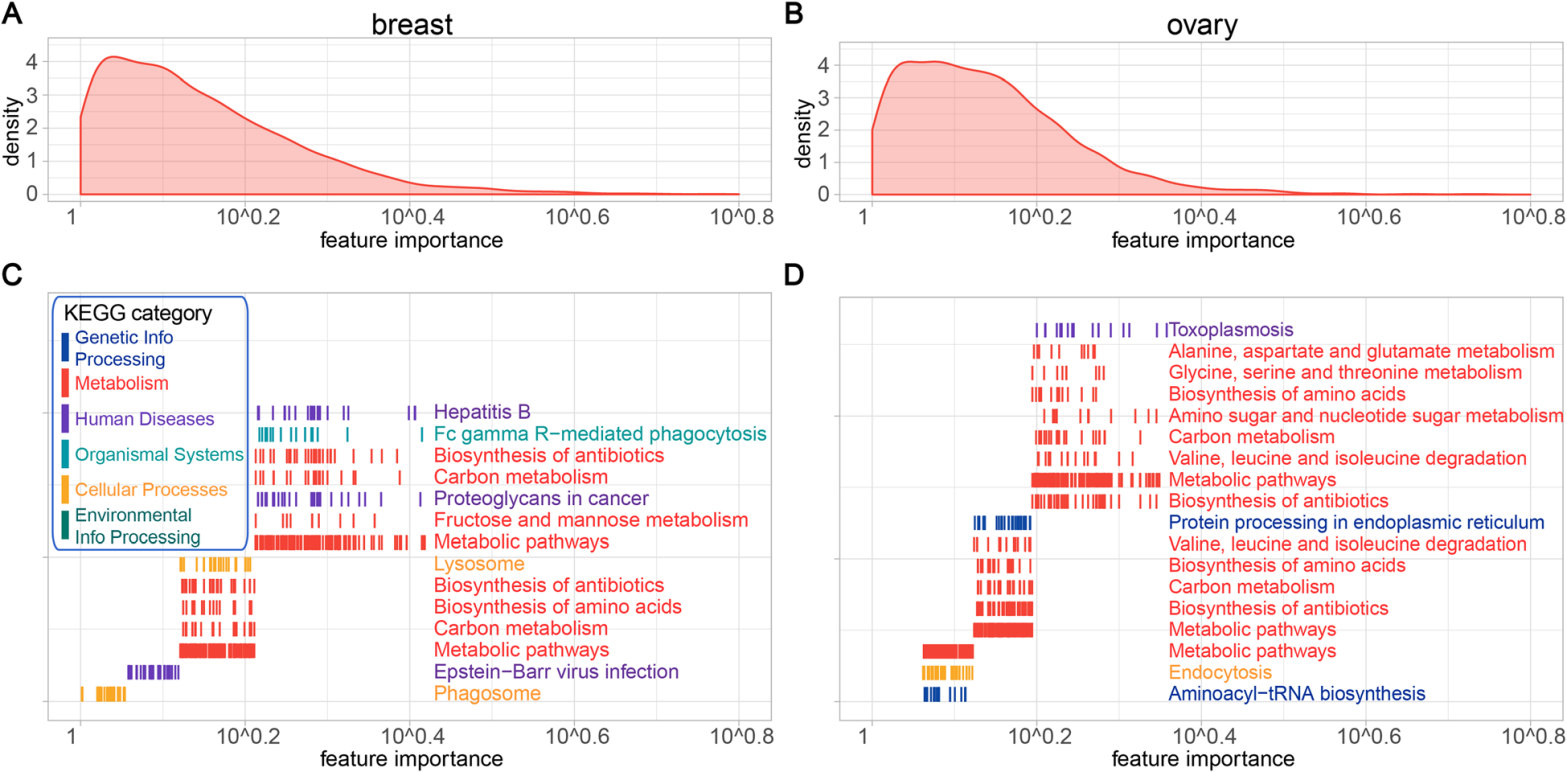
The functional enrichment analysis of gene sets that drive the regulation of protein abundance. **a**, **b** The overall distribution of the gene importance in predicting protein abundance are shown in **a** breast and **b** ovarian cancers. **c**, **d** Functional enrichment analysis was performed on gene subsets based on the feature importance. The colors represent the major KEGG categories. Genes that play more important roles in regulating protein abundance are mainly associated with “Metabolism”

Metabolism-related gene sets make major contributions to predicting protein abundance. To evaluate the feature importance of a gene, the mRNA values of each gene across samples were permuted and the prediction performance was re-evaluated. Permutation of more important genes resulted in larger drops in performances, which were considered as the feature importance (Additional file 2: Table S8–S9). Based on the importance, we ranked all genes and performed functional enrichment analysis on the important “driver” genes. We found that genes of the KEGG “Metabolism” category played an essential role (Fig. 5). As we expected, among pathways of carbon metabolism, biosynthesis of amino acids was more critical in determining the protein abundance.

**Fig. 6:**
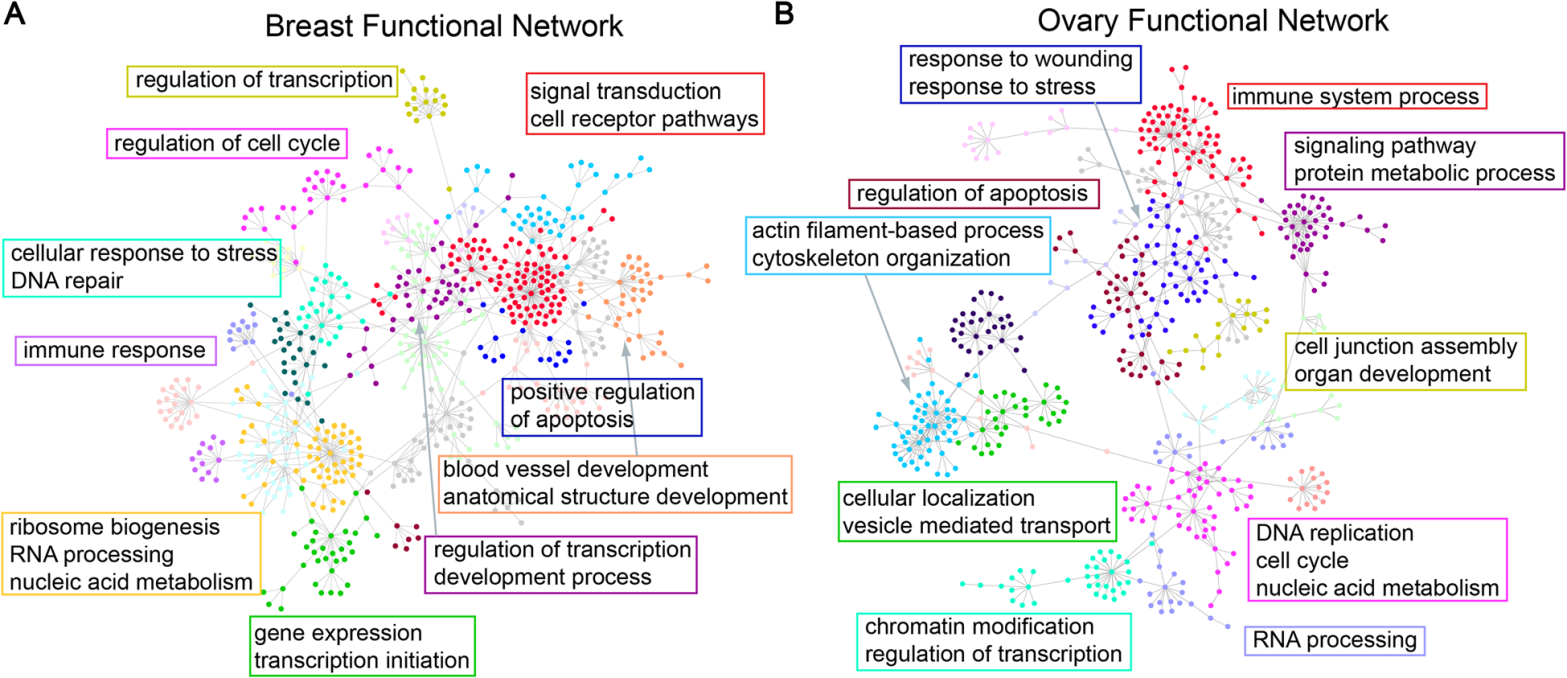
Functional clusters in the gene-gene interaction network that drive the regulation of protein abundance. **a**, **b** Decomposition of gene functional network among “driver” genes in breast (**a**) and ovarian (**b**) cancers reported important metabolism pathways. The gene clusters were shown in different colors and visualized using a gene-gene interaction network. The shared biological processes of selected clusters were labeled in rectangles

To further investigate these “driver” genes, we mapped them to a gene functional network [23–25]. This network was constructed based on a Bayesian integration of diverse genetic and functional genomic data. We extracted a subnetwork that contained only the driver genes as well as edges that had high estimated probability of the co-functioning relationship (Fig. 6). The high-confidence connections encompassed 674 “driver” and “target” genes in ovarian cancer and 568 in breast cancer (Additional file 2: Table S10–S11). Then, we applied the Girvan-Newman community clustering algorithm to the subnetwork. The algorithm iteratively identifies and cuts the sparse connections that connect different modules to maximize a modularity score [26, 27].

The resulting clusters are a collection of gene modules that are highly connected within the cluster but loosely connected to other genes. The GO term enrichment analysis was further performed on the resulting modules. The important enriched pathways fell into a number of naturally forming groups. Specifically, the processes of gene expression, protein metabolics, transcription initiation, and regulation were enriched. The initiation of protein translation is known to be the bottleneck step of the protein synthesis [28]. The pathways of cell cycle regulation and DNA/RNA modification were also prominently featured. Additionally, the immune response, signal transduction, response to wounds, and morphological development were all enriched. Interestingly, it has been reported that cellular stress responses and wound healing are related to cancer treatment resistance and metastasis [29–31]. The results confirmed our expectation that the nexus modules formed by these genes are loosely but confidently associated with other genes. The translation level of a protein is controlled by a complex network consisting of diverse regulatory elements in the cells.

## Discussion

From the central dogma to the complex protein functional networks and pathways, our understanding of protein expression regulation has been revolutionized over the past 60 years. Although macromolecular interactions require specific physicochemical interfaces [32], indirect interactions and high-level associations exist in cellular environment. In terms of predicting protein abundance from transcriptomic data, these ubiquitous associations among all genes play an indispensable role. This indicates that in addition to the idea of functional pathways and protein-protein interaction networks, considering the general direct and indirect interactions among all genes is a complement towards understanding the underlying mechanisms [33]. In addition, we found that adding amino acid composition and protein class as features improved prediction. A recent study also leveraged sequence-based features to predict protein-to-mRNA ratios, including 5′ untranslated region (UTR) folding energy, coden frequency, and 5′ end hydrophobicity [34]. We envision that these sequence-based features are potentially helpful in future development of machine learning models for predicting proteomes in cancer patients.

Technical advances in the past two decades have enabled us to investigate the quantitative relationship between the concentration of a transcript and the concentration of a protein through transcriptomic and proteomic profilings. There are two typical ways of calculating the correlation between predictions and observations [7]. The first way is calculating the correlation across genes within the same sample [8]. In previous studies, the protein-to-mRNA ratios were first estimated for each gene. Then, the protein level was predicted through multiplying the mRNA level by the gene-specific protein-to-mRNA ratio [35, 36]. However, this type of correlation cannot answer the key question about protein expression regulation—to what extent the variation of mRNA levels influences the corresponding protein level [7]. In addition, it has been clearly pointed out that this within-sample correlation was dramatically overestimated [37]. The second way is calculating the within-gene correlation across samples. In addition to the understanding of protein expression regulation, the cross-sample proteome prediction is especially useful in cancer research (e.g., for distinguishing between high-risk and low-risk cancer patients) [10, 11]. In the NCI-CPTAC Proteogenomics DREAM Challenge, the predictive performance was evaluated using the cross-sample correlation, instead of the within-sample correlation. Our joint training approach ranked first in predicting both breast and ovarian cancer proteomes, outperforming other methods including random forest with features derived from KEGG pathways [18] and Human Protein Reference Database (HPRD) [38] and LASSO regression with features derived from Protein-Protein interaction network (BioGRID) [39] and Protein complex network (CORUM) [40].

Many pioneering efforts have been made to characterize the proteogenomic features of various cancers [10–12, 41]. However, how to integrate information from multiple cancers to foster cancer research remains unclear. In this study, we propose a simple yet effective attempt to address this problem, facilitating the prediction of protein abundance. It would be interesting to see where the information is shareable among diverse cancers or other diseases, beyond breast and ovarian cancers. Intriguingly, we observe that protein subsets that are hard to predict are enriched in several neural degenerative diseases.

The ideas of training models across two cancer tissues are inspired by the transfer learning strategy widely used in recent deep learning tasks, including computer vision and natural language processing. Transfer learning refers to the situation when a model is trained on one task and used/adapted on another related task [42]. In this work, we have one task of predicting protein abundance but we need to build models for two cancer tissues. We applied a joint learning strategy to build the trans-tissue model, which is similar to transfer learning in a broad sense.

## Conclusions

We present a novel method to predict protein abundance by transferring information across tissues. The ideas embedded in our approach, including general gene-gene associations and information transfer across cancer types, will provide useful insights into protein expression regulation and cancer research.

## Methods

### Data collection

For both breast and ovarian cancers, the proteome data were acquired using the isobaric Tags for Relative and Absolute Quantification protein quantification method. The proteomics data were downloaded from CPTAC data portal. For breast proteome, 77 samples were analyzed at the Broad Institute (BI). For ovarian proteome, 84 and 122 samples were analyzed at Pacific Northwest National Laboratory (PNNL) and Johns Hopkins University (JHU), respectively. The protein log ratios of the protein abundance were calculated including only peptides that map unambiguously to the protein. Only 105 samples from different patients with TCGA RNA-seq data were used in this work. The transcriptomics data for the corresponding breast and ovarian cancer samples were downloaded from TCGA firehose.

### Proteomic data processing

The proteomic data were processed by the standard data analysis pipeline from CPTAC, which was described in detail in the original CPTAC publications [11, 12]. The tumor sample tissues were first digested into peptide with trypsin and the digested samples were labeled with 4-plex iTRAQ [43]. These labeled samples were subsequently fractionated by basic reversed phase liquid chromatography (LC) to reduce sample complexity. The LC separated samples were used in the LC-MS/MS system for proteome analysis. MS analysis was performed using the Thermo Scientific LTQ Orbitrap Velos mass spectrometer. Thermo RAW files were processed with DeconMSn (v2.3.0) to determine the m/z values and charge of the precursor ions and saved as CDTA files. Then, the CDTA files were processed with DTARefinery [44] to correct for instrument calibration errors. The database search engine MSGF+ [45] was used to match the CDTA files against the RefSeq human protein sequence database (release version 37). Subsequently, peptides were identified and assembled into proteins using IDPicker [46]. The maximum false discovery rate (FDR) was set to 1% at peptide level, and a minimum of 3 unique peptides was required to identify a protein. The intensities of iTRAQ reporter ions were extracted using MASIC [47] and the ratio of sample abundance to reference abundance from ion intensities was calculated as the relative protein abundance. The relative abundances were further log2 transformed to obtain the relative expression values. To reduce the sample-specific bias, the protein abundances from the same sample were re-centered to achieve a common median of zero.

### Transcriptomic data processing

The transcriptomic data were processed by the standard data analysis pipeline from TCGA, which was described in detail in the original publication [48]. The raw RNA sequencing data were generated using the Illumina HiSeq, and the sequencing reads were aligned to the human reference genome (hg19) using MapSlice [49]. The gene expression levels were first quantified for the transcript models (TCGA GAF 2.13) using RSEM4 [50] and normalized to a fixed upper quantile within each sample. Then, the normalized data were further log2 transformed and median centered by gene. If a gene had a value of zero before log2 transformation, then this gene was labeled by “NA” (missing value) after log2 transformation.

### Generic model

For each gene *i*, the mRNA levels across patients were used as the baseline predictions for the corresponding protein abundance across the same patients (top-left in Fig. 1). If the mRNA values were missing, we used the average of all non-missing RNA observations of the same gene as the imputation:

$$ {x}_{\mathrm{missing}}=\left(\;\sum \limits_{i=0}^{n_{\mathrm{non}-\mathrm{missing}}}{x}_i\;\right)/{n}_{\mathrm{non}-\mathrm{missing}} $$

where *x*_*i*_ represents the mRNA level of a non-missing sample and *n*_non-missing_ represents the number of non-missing samples.

### Gene-specific model

The entire RNA-seq data is represented by a *m*-by-*n* matrix X,

$$ \left[\begin{array}{cccc}x11& x12& \cdots & x1n\\ {}x21& x22& \cdots & x2n\\ {}\vdots & \vdots & \ddots & \vdots \\ {} xm1& \cdots & \cdots & xmn\end{array}\right] $$

where rows represent genes and columns represent samples. An element *x*_*ij*_ denotes the mRNA level of gene *i* from sample *j*. Similar to mRNA, the proteomic data is represented by a *s*-by-*n* matrix Y,

$$ \left[\begin{array}{cccc}y11& y12& \cdots & y1n\\ {}y21& y22& \cdots & y2n\\ {}\vdots & \vdots & \ddots & \vdots \\ {} ys1& \cdots & \cdots & ysn\end{array}\right] $$

where rows represent proteins and columns represent samples. For each gene, we created a gene-specific random forest (RF) model [51], with a maximum depth of 3 and 100 trees (bottom in Fig. 1). As one of the tree-based models, RF has been reported to avoid overfitting and capture nonlinear interactions between features [52–55]. For example, for gene *i*, we treated the protein levels of this gene across *n* samples (*y*_*i*1_, *y*_*i*2_, …, *y*_*in*_) as *n* targets. For each sample *y*_*ik*_, we use its corresponding mRNA levels of all *m* genes (*x*_1*k*_, *x*_2*k*_, …, *x*_*mk*_) as a vector of *m* features. In this way, we trained a model using *n* samples. And for a different gene *j*, we created a different model since the target values across *n* samples (*y*_*j*1_, *y*_*j*2_, …, *y*_*jn*_) are different. Thus, we call this a gene-specific model. After excluding genes with missing mRNA values, the total numbers of feature genes are 8738 and 5837 in breast and ovarian cancers, respectively. These models were implemented using the function called ensemble.RandomForestRegressor of python module scikit learn.

### Trans-tissue model

The numbers of proteins to be predicted are 10,006 and 7061 in breast and ovarian cancers, respectively. Among them, 6934 proteins are common in the two cancers. To pool regulatory information between two cancers, we combined the patient samples for each common protein and trained the trans-tissue random forest model in the same way as the gene-specific model (top-right in Fig. 1). The total number of training samples is 182 (77 breast and 105 ovarian).

### Statistical analysis

To compare the prediction correlations among different models, the bootstrap sampling with replacement was performed. Specifically, 5000 genes were randomly selected to calculate the overall prediction correlation of a model in each bootstrap sample. The sampling was performed 1000 times for each model, followed by the Wilcoxon signed-rank test to compare two models. The differences between all pairs of models in Fig. 2a, b were statistically significant (*p* < 2.2e−16). The *p* values were calculated using the default function wilcox.test in R version 3.4.4.

### Fivefold cross validation

To systematically compare the performance of different models and features, fivefold cross validation was performed on the training data of 77 breast and 105 ovarian cancer samples. For each cancer, the entire training samples were randomly partitioned into five non-overlapping subsets. In each validation, four subsets were used to train a model and one subset was used to validate the performance of this model. This resulted in 5 scores, reflecting the overall performance of a model on the entire dataset.

### Comparing models using different numbers of features

To evaluate the effects of using different numbers of features, the top 10, 100, and 1000 highly expressed genes and all genes (8738 breast genes and 5837 ovarian genes) were used to train the gene-specific models. We further evaluated the filtered gene subset based on GO terminology (GO 0010467: gene expression and GO 0010468: regulation of gene expression), resulting in 4472 and 4473 feature genes in the GO breast and ovarian cancer models.

### Comparing models trained on different numbers of samples

To evaluate the effects of training different numbers of samples, 20%, 40%, 80%, and 100% of training samples were randomly selected to train the gene-specific model. Then, the samples from the breast and ovarian cancers were combined and trained in the trans-tissue model.

### Model ensemble

For each protein, the weighted average predictions from the generic and the gene-specific models were calculated, with the weighting ratio of 1:3. For the 6934 common proteins, the predictions from the trans-tissue model were added, with the weighting ratio of 1:1. It should be noted that, for non-common proteins, the trans-tissue model is not applicable. These weights were used to generate predictions. To evaluate the effect of different weighting ratios, we performed a grid-search of all possible weights from 0 to 10 among the generic, gene-specific, and trans-tissue models.

### Evaluation metrics

To evaluate the performance of different models, Pearson’s correlation between observed and predicted abundances across all samples was calculated for each protein. We then took the mean correlations of all proteins as the primary evaluation score. In addition, the normalized root mean square error (NRMSE) was used as the secondary metric to compare models.

The formula for computing the Pearson correlation *r* is as follows:

$$ r=\frac{1}{n_{\mathrm{obs}}-1}\sum \limits_{i=1}^{n_{\mathrm{obs}}}\ \frac{\left({x}_i-\underset{\_}{x}\right)\left({y}_i-\underset{\_}{y}\right)\ }{S_x\times {S}_y} $$

The formula for computing NRMSE is as follows:

$$ \mathrm{NRMSE}=\frac{\sqrt{\sum_{i=1}^{n_{\mathrm{obs}}}{\left({y}_i-{x}_i\right)}^2/{n}_{\mathrm{obs}}}}{y_{\mathrm{max}}-{y}_{\mathrm{min}}} $$

The observed and predicted values are denoted by *y* and *x*, respectively. *S*_*y*_ and *S*_*x*_ are their standard deviations. For each protein, *n*_obs_ is the number of observed samples. And *y*_max_ and *y*_min_ are the respective maximal and minimal value across all observed samples.

### Correlations and RMSEs between experimental replicates

There were 32 overlapping ovarian cancer samples measured at both JHU and PNNL. These overlapping samples were used to estimate the theoretical best performance that could be achieved by a computational prediction method. Pearson’s correlations and RMSEs for all 5218 proteins under consideration were calculated across the 32 ovarian cancer samples.

### Feature importance

Random forest enables us to estimate the importance of each chemical feature by permuting the values of a feature across samples and computing the increase in prediction error, delta-error. More important feature genes have larger delta-error. Based on the delta-error, we evaluate the importance of all feature genes.

### Functional enrichment analysis

All the evaluated proteins were quantile partitioned into four subsets based on the prediction performance. For each subset, functional annotation was performed using DAVID. We further analyzed the functional enrichment of proteins ranked by the improvement compared with the baseline mRNA and protein levels and proteins playing important roles in regulating the protein abundance of all genes.

### Functional network analysis

The top 500 genes with the highest feature importance (“driver” genes) were mapped to a gene functional network. A subset of highly connected genes were selected for the clustering analysis (674 genes in the breast and 568 genes in the ovary). These genes, together with edges among these genes, were extracted to a subnetwork. The network was then fed into GLay community clustering method. The clustering method is based on the Girvan-Newman algorithm [23] and implemented in ClusterMaker2, a Cytoscape plugin. The method dissects the original subnetwork into multiple modules. Each of the modules was then fed into BINGO, a Cytoscape plugin, for GO term enrichment analysis.

### Figure preparation

The figures were prepared using R package ggplot2, ggtern and GGally. The protein structures shown as a 3D illustration in Fig. 1 are downloaded from Protein Data Bank. Their IDs are 1cr5, 1ctq, 1grn, 1jbb, 1kpc, 1tnd, 1yfp, and 1zho. These images were generated by VMD 1.9.3.

### Availability of code

Source code: https://github.com/GuanLab/proteome_prediction

## Supplementary information

**Additional file 1: **Extended methods and supplementary figures. **Figure S1.** The distribution of correlations in breast. **Figure S2.** The distribution of correlations in ovary. **Figure S3.** The RMSEs of different models. **Figure S4.** The distribution of RMSEs in breast. **Figure S5.** The distribution of RMSEs in ovary. **Figure S6.** Correlations using different numbers of genes as features. **Figure S7.** The distribution of correlations using different numbers of features in breast. **Figure S8.** The distribution of correlations using different numbers of features in ovary. **Figure S9.** RMSEs using different numbers of genes as features. **Figure S10.** The distribution of RMSEs using different numbers of features in breast. **Figure S11.** The distribution of RMSEs using different numbers of features in ovary. **Figure S12.** The distribution of correlations of different models in breast. **Figure S13.** The distribution of correlations of different models in ovary. **Figure S14.** The distribution of RMSEs of different models in breast. **Figure S15.** The distribution of RMSEs of different models in ovary. **Figure S16.** Correlations using different numbers of samples. **Figure S17.** The distribution of correlations using different numbers of samples in breast. **Figure S18.** The distribution of correlations different numbers of samples in ovary. **Figure S19.** RMSEs using different numbers of samples. **Figure S20.** The distribution of RMSEs using different numbers of samples in breast. **Figure S21.** The distribution of RMSEs using different numbers of samples in ovary. **Figure S22.** The effects of different training scenarios and normalization strategies. **Figure S23.** The correlation comparison with experimental replicates. **Figure S24.** The pair-wise correlation comparison with experimental replicates. **Figure S25.** The RMSE comparison with experimental replicates. **Figure S26.** The pair-wise RMSE comparison with experimental replicates. **Figure S27.** The functional enrichment analysis of gene sets with different correlation increases.

**Additional file 2: **Supplementary Tables. **Table S1.** The five-fold Pearson’s correlations of the generic, gene-specific and trans-tissue models. **Table S2.** The five-fold correlations of models using different numbers of features. **Table S3.** The weighting ratios to stack the generic, gene-specific and trans-tissue models and the corresponding prediction correlations in breast. **Table S4.** The weighting ratios to stack the generic, gene-specific and trans-tissue models and the corresponding prediction correlations in ovary. **Table S5.** The five-fold correlations of models using different numbers of samples. **Table S6.** The correlations and RMSEs across 32 overlapping ovarian cancer samples measured at both JHU and PNNL. **Table S7.** The correlations and RMSEs of our predictions on the held-out testing dataset of 82 ovarian cancer samples during the NCI-CPTAC DREAM challenge. **Table S8.** The feature importance of all genes in breast. **Table S9.** The feature importance of all genes in ovary. **Table S10.** The list of driver genes and their clusters in the breast functional network. **Table S11.** The list of driver genes and their clusters in the ovary functional network.

## Data Availability

All data generated or analyzed during this study are included in this published article and its supplementary information files. The raw data used in this study are accessible from NCI-CPTAC and TCGA data portal: (i) breast cancer proteomic and phosphoproteomic data [56], (ii) ovarian cancer proteomic and phosphoproteomic data [57], (iii) breast cancer genomic data [58], and (iv) ovarian cancer genomic data [59]. The challenge dataset repository is accessible from the synapse website [60].

## Abbreviations

NCI: National Cancer Institute
CPTAC: Clinical Proteomic Tumor Analysis Consortium
TCGA: The Cancer Genome Atlas
DREAM: Dialogue on Reverse Engineering Assessment and Method
RF: Random forest
NRMSE: Normalized root mean square error
GO: Gene Ontology
CNV: Copy number variation
KEGG: Kyoto Encyclopedia of Genes and Genomes
