# Supplementary material for: Joint learning improves protein abundance prediction in cancers
Source: BMC Biol. 2019 Dec 23;17:107. doi: 10.1186/s12915-019-0730-9 (PMC6929375; doi:10.1186/s12915-019-0730-9)
Supplement: Supplementary file 1 — Additional file 1: Extended methods and supplementary figures. Figure S1. The distribution of correlations in breast. Figure S2. The distribution of correlations in ovary. Figure S3. The RMSEs of different models. Figure S4. The distribution of RMSEs in breast. Figure S5. The distribution of RMSEs in ovary. Figure S6. Correlations using different numbers of genes as features. Figure S7. The distribution of correlations using different numbers of features in breast. Figure S8. The distribution of correlations using different numbers of features in ovary. Figure S9. RMSEs using different numbers of genes as features. Figure S10. The distribution of RMSEs using different numbers of features in breast. Figure S11. The distribution of RMSEs using different numbers of features in ovary. Figure S12. The distribution of correlations of different models in breast. Figure S13. The distribution of correlations of different models in ovary. Figure S14. The distribution of RMSEs of different models in breast. Figure S15. The distribution of RMSEs of different models in ovary. Figure S16. Correlations using different numbers of samples. Figure S17. The distribution of correlations using different numbers of samples in breast. Figure S18. The distribution of correlations different numbers of samples in ovary. Figure S19. RMSEs using different numbers of samples. Figure S20. The distribution of RMSEs using different numbers of samples in breast. Figure S21. The distribution of RMSEs using different numbers of samples in ovary. Figure S22. The effects of different training scenarios and normalization strategies. Figure S23. The correlation comparison with experimental replicates. Figure S24. The pair-wise correlation comparison with experimental replicates. Figure S25. The RMSE comparison with experimental replicates. Figure S26. The pair-wise RMSE comparison with experimental replicates. Figure S27. The functional enrichment analysis of gene sets with different correlation increase [file 12915_2019_730_MOESM1_ESM.docx]

# **Supplemental Information**

**Joint Learning Improves Protein Abundance Prediction in Cancers**

Hongyang Li^1,*^, Omer Siddiqui^1^ , Hongjiu Zhang^1^, Yuanfang Guan^1,2,*^

1. Department of Computational Medicine and Bioinformatics, University of Michigan, 100 Washtenaw Avenue, Ann Arbor, MI 48109, USA

2. Department of Internal Medicine, University of Michigan, 100 Washtenaw Avenue, Ann Arbor, MI 48109, USA

* Corresponding author: [gyuanfan@umich.edu](mailto:gyuanfan@umich.edu)**,** [hyangl@umich.edu](mailto:hyangl@umich.edu)

**Contents**

**Extended Methods**

**Five-fold cross validation** ……………………………………………………..…………... 2

**Comparing models using different numbers of features** ……………….…………… 2

**Comparing models trained on different numbers of samples** …………….………... 2

**Model ensemble** ………………...………………………………………….………...……... 2

**Evaluation metrics** ……………………………………………...………….…………...…... 3

**Correlations and RMSEs between experimental replicates** ……………….………... 3

**Feature importance** ……………………………………………………………….…….…... 3

**Functional enrichment analysis** …………………………………...………….………….. 3

**Functional network analysis** ……………………………………….…………….……….. 3

**Figure preparation** ……………………………………………………..……….…………... 4

**Supplementary Figures**

**Supplementary Figure 1** …….………….…………………….………..………………….... 5

**Supplementary Figure 2** …….………….……………………………...………………….... 6

**Supplementary Figure 3** …….………….………………………………...……………….... 7

**Supplementary Figure 4** …….………….………………………………………………….... 8

**Supplementary Figure 5** …….………….……………………………...………………….... 9

**Supplementary Figure 6** ………………………………………………...……………….... 10

**Supplementary Figure 7** ………………………………………………………………….... 11

**Supplementary Figure 8** ……………………………………………...………………….... 12

**Supplementary Figure 9** ………………………………………………...……………….... 13

**Supplementary Figure 10** ………………………………….…………………………….... 14

**Supplementary Figure 11** …………………………………………...……………..…….... 15

**Supplementary Figure 12** ……………………………………………...……..………….... 16

**Supplementary Figure 13** ………………………………………..……...……………….... 17

**Supplementary Figure 14** …………………………………………...…..……………….... 18

**Supplementary Figure 15** …………………………………..…………...……………….... 19

**Supplementary Figure 16** …………………………………………….………………….... 20

**Supplementary Figure 17** ………………………………….…………...……………….... 21

**Supplementary Figure 18** ………………………………….……………...…………….... 22

**Supplementary Figure 19** ………………………………………………….…………….... 23

**Supplementary Figure 20** ……………………………….……………...……………….... 24

**Supplementary Figure 21** ………………………………….……………...…………….... 25

**Supplementary Figure 22** …………………………………………………….………….... 26

**Supplementary Figure 23** ……………………………………………...……………….... 27

**Supplementary Figure 24** ……………………………………………………………….... 28

**Supplementary Figure 25** ……………………………………………………………….... 29

**Supplementary Figure 26** ……………………………………………………………….... 30

**Supplementary Figure 27** ……………………………...……………………………….... 31

**Supplementary Table Legends** …………………..…………………………..……………..….... 32

**Extended Methods**

**Five-fold cross validation**

To systematically compare the performance of different models and features, five-fold cross validation was performed on the training data of 77 breast and 105 ovarian cancer samples. For each cancer, the entire training samples were randomly partitioned into 5 non-overlapping subsets. In each validation, 4 subsets were used to train a model and 1 subset was used to validate the performance of this model. This resulted in 5 scores, reflecting the overall performance of a model on the entire dataset.

**Comparing models using different numbers of features**

To evaluate the effects of using different number of features, the top 10, 100, and 1000 highly expressed genes, and all genes (8738 breast genes and 5837 ovarian genes) were used to train the gene-specific models. We further evaluated the filtered gene subset based on GO terminology (GO 0010467: gene expression and GO 0010468: regulation of gene expression), resulting in 4472 and 4473 feature genes in the GO breast and ovarian cancer models.

**Comparing models trained on different numbers of samples**

To evaluate the effects of training different numbers of samples, 20%, 40%, 80% and 100% of training samples were randomly selected to train the gene-specific model. Then the samples from the breast and ovarian cancers were combined and trained the trans-tissue model.

**Model ensemble**

For each protein, the weighted average predictions from the generic and the gene-specific models were calculated, with the weighting ratio of 1:3. For the 6934 common proteins, the predictions from the trans-tissue model were added, with the weighting ratio of 1:1. It should be noted that, for non-common proteins, the trans-tissue model is not applicable. These weights were used to generate predictions. To evaluate the effect of different weighting ratios, we performed a grid search of all possible weights from 0 to 10 among the generic, gene-specific and trans-tissue models.

**Evaluation metrics**

To evaluate the performance of different models, the Pearson’s correlation between observed and predicted abundances across all samples was calculated for each protein. We then took the mean correlations of all proteins as the primary evaluation score. In addition, the Normalized Root Mean Square Error (NRMSE) was used as the secondary metric to compare models.

The formula for computing the Pearson correlation r, is as follow:

$$r=\frac{1}{n_{obs}-1}\sum_{i=1}^{n_{obs}} \frac{(x_{i}-\underline{x})(y_{i}-\underline{y})}{S_{x}S_{y}}$$

The formula for computing NRMSE is as follows:

$$NRMSE=\frac{\sqrt{\sum_{i=1}^{n_{obs}} (y_{i}-x_{i})^{2}/n_{obs}}}{y_{max}-y_{min}}$$

The observed and predicted values are denoted by y and x, respectively. S_y_ and S_x_ are their standard deviations. For each protein, nobs is the number of observed samples. And y_max_ and y_min_ are the respective maximal and minimal value across all observed samples.

**Correlations and RMSEs between experimental replicates**

There were 32 overlapping ovarian cancer samples measured at both JHU and PNNL. These overlapping samples were used to estimate the theoretical best performance that could be achieved by a computational prediction method. The Pearson’s correlations and RMSEs for all 5,218 proteins under consideration were calculated across the 32 ovarian cancer samples.

**Feature importance**

Random forest enables us to estimate the importance of each chemical feature by permuting the values of a feature across samples and computing the increase in prediction error, delta-error. More important feature genes have larger delta-error. Based on the delta-error, we evaluate the importance of all feature genes.

**Functional enrichment analysis**

All the evaluated proteins were quantile partitioned into four subsets based on the prediction performance. For each subset, functional annotation was performed using DAVID. We further analyzed the functional enrichment of proteins ranked by the improvement compared with the baseline mRNA and protein levels, and proteins playing important roles in regulating the protein abundance of all genes.

**Functional network analysis**

The top 500 genes with the highest feature importance (“driver” genes) were mapped to a gene functional network. A subset of highly connected genes were selected for the clustering analysis (674 genes in breast and 568 genes in ovary). These genes, together with edges among these genes, were extracted to a subnetwork. The network was then fed into GLay community clustering method. The clustering method is based on the Girvan-Newman algorithm [[23]](https://paperpile.com/c/ZVSvI2/oDrdn) and implemented in ClusterMaker2, a Cytoscape plugin. The method dissects the original subnetwork into multiple modules. Each of the modules was then fed into BINGO, a Cytoscape plugin, for GO term enrichment analysis.

**Figure preparation**

The figures were prepared using R package ggplot2, ggtern and GGally. The protein structures shown as 3D illustration in Figure 1 were downloaded from Protein Data Bank. Their IDs are 1cr5, 1ctq, 1grn, 1jbb, 1kpc, 1tnd, 1yfp and 1zho. These images were generated by VMD 1.9.3.

**
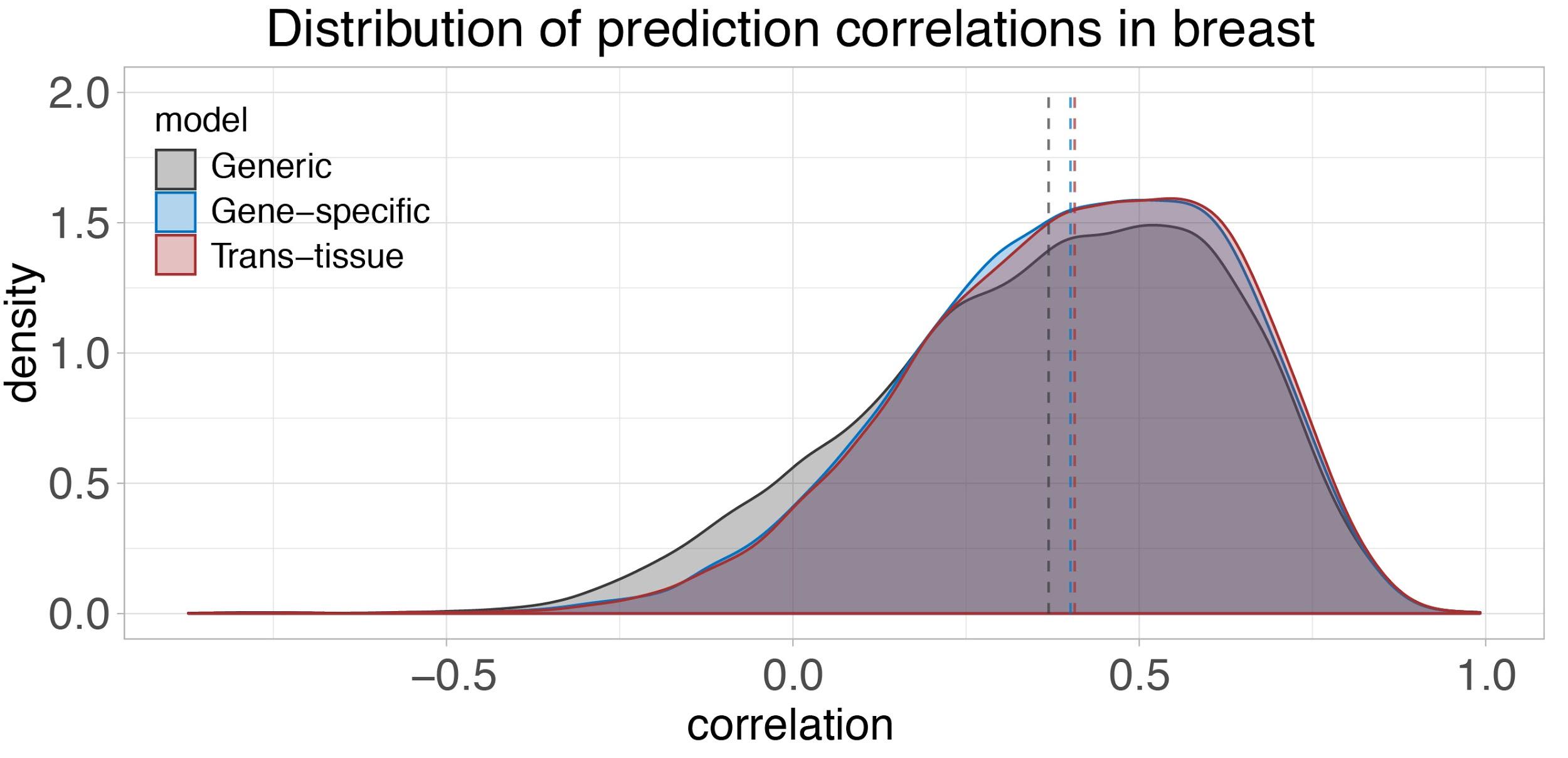
**

**Supplementary Figure 1. The distribution of correlations between predictions and observations across samples for all proteins in breast.**

The “Generic”, “Gene-specific” and “Trans-tissue” models are shown in grey, blue and red, respectively. The dashed line represents the average correlation of all proteins.


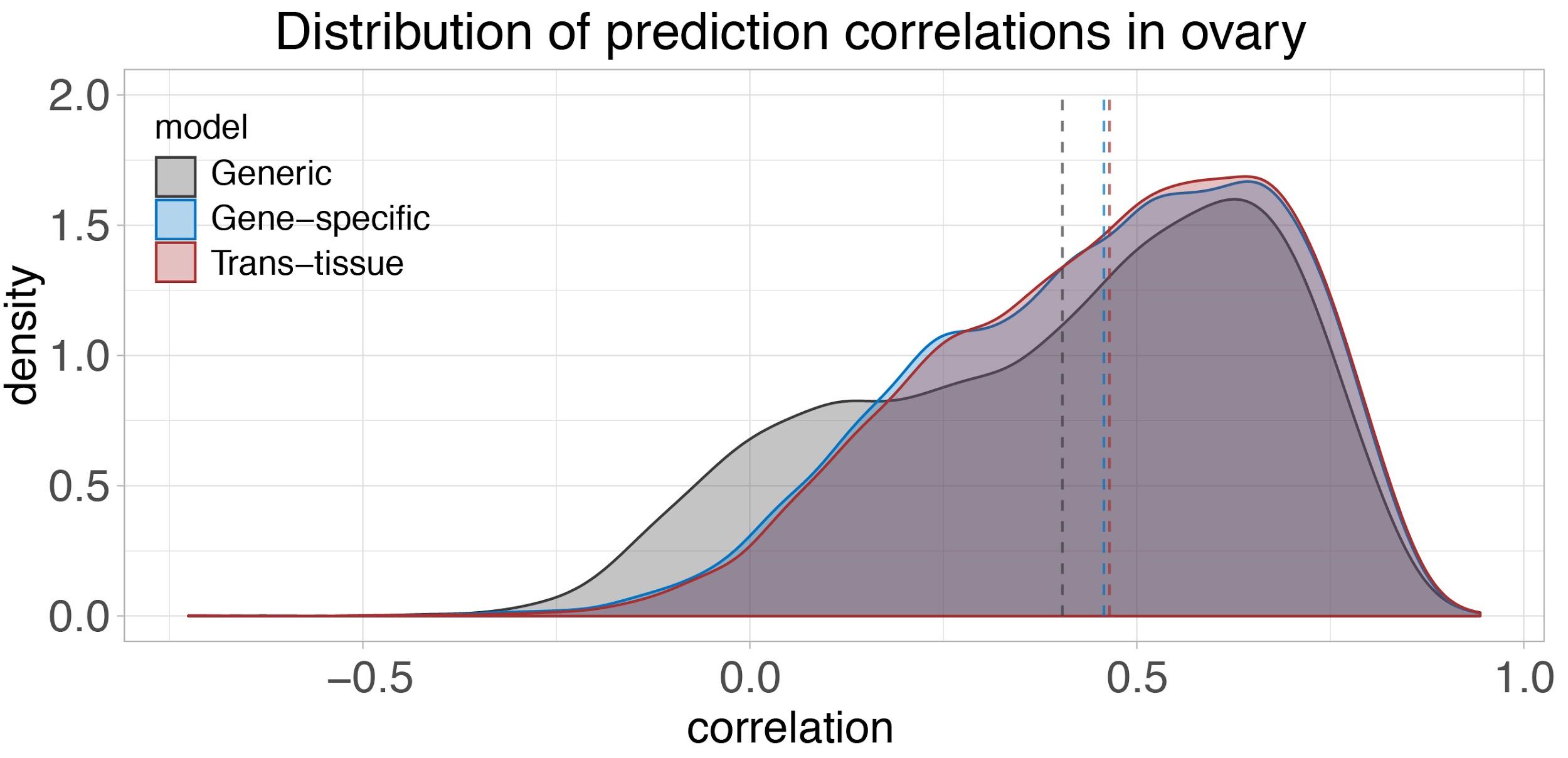


**Supplementary Figure 2. The distribution of correlations between predictions and observations across samples for all proteins in ovary.**

The “Generic”, “Gene-specific” and “Trans-tissue” models are shown in grey, blue and red, respectively. The dashed line represents the average correlation of all proteins.


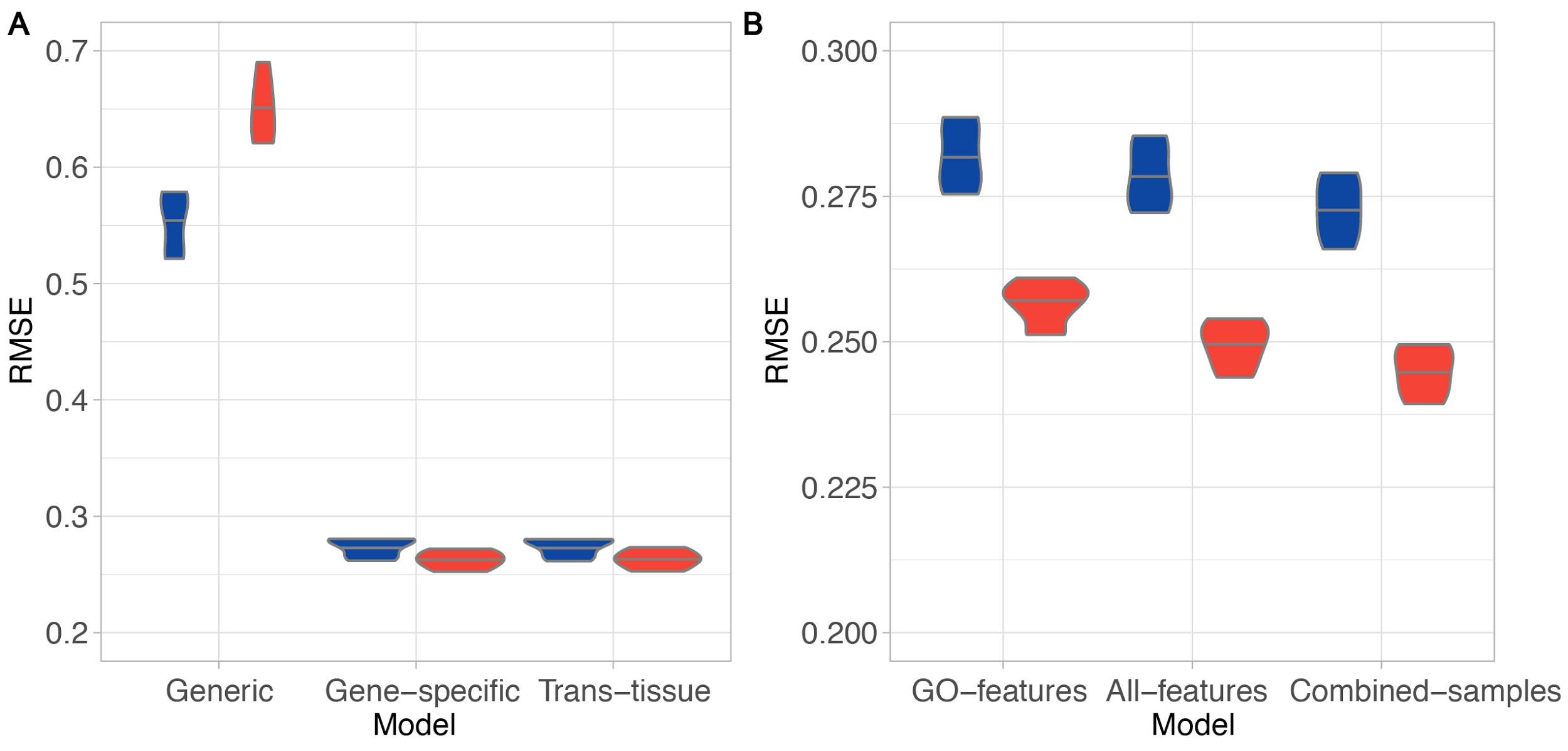


**Supplementary Figure 3. The RMSEs of different models in predicting proteome in breast and ovarian cancers.**

**A.** From left to right, the RMSEs were calculated by assembling the following three models step by step (blue: breast; red: ovary): 1) The generic model, which only uses the transcript-level expression of a target protein as the only feature; 2) The gene-specific model, which uses the transcript-level expressions of all genes as features for predicting a target protein; 3) The trans-tissue model, which is similar to the gene-specific model yet combines both breast and ovarian cancer samples. **B.** Dissection of the gene-specific model by using different sets of features and samples. 1）Sub-selecting all genes related to ‘gene expression’ as features. 2）Using all transcripts as features to predict the target protein. 3) Combining samples from two tissues to train.


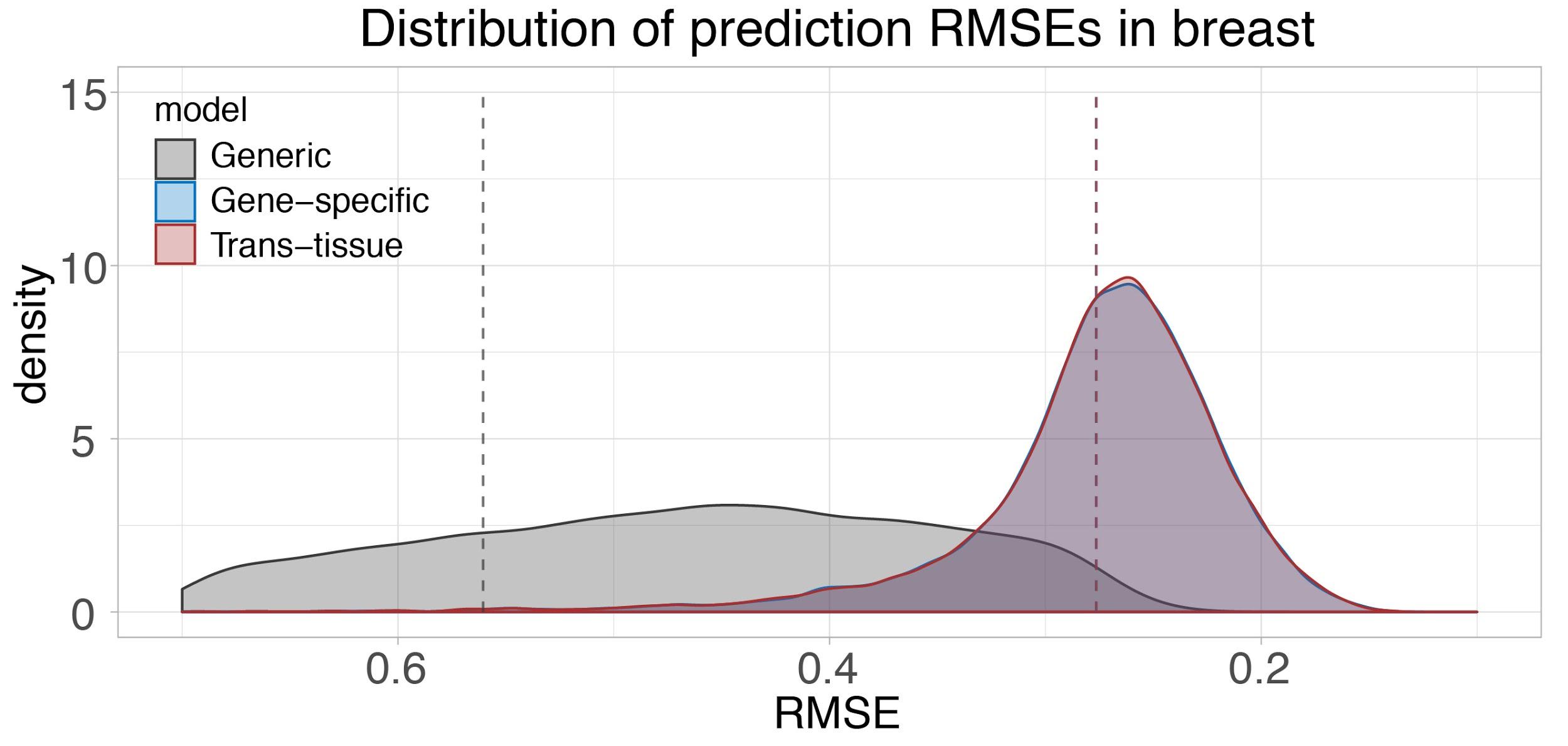


**Supplementary Figure 4. The distribution of RMSEs between predictions and observations across samples for all proteins in breast.**

The “Generic”, “Gene-specific” and “Trans-tissue” models are shown in grey, blue and red, respectively. The dashed line represents the average RMSe of all proteins.


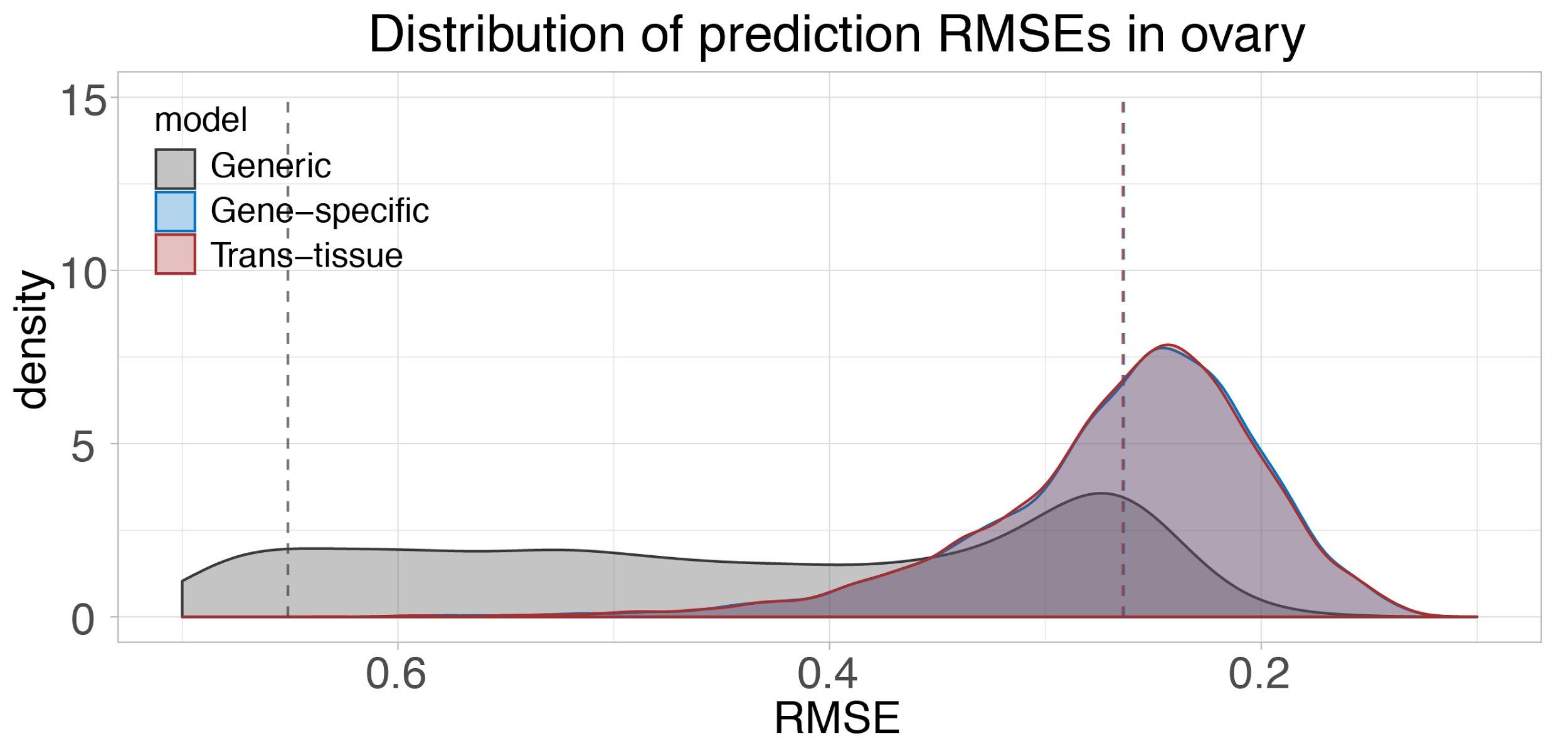


**Supplementary Figure 5. The distribution of RMSEs between predictions and observations across samples for all proteins in breast.**

The “Generic”, “Gene-specific” and “Trans-tissue” models are shown in grey, blue and red, respectively. The dashed line represents the average RMSE of all proteins.


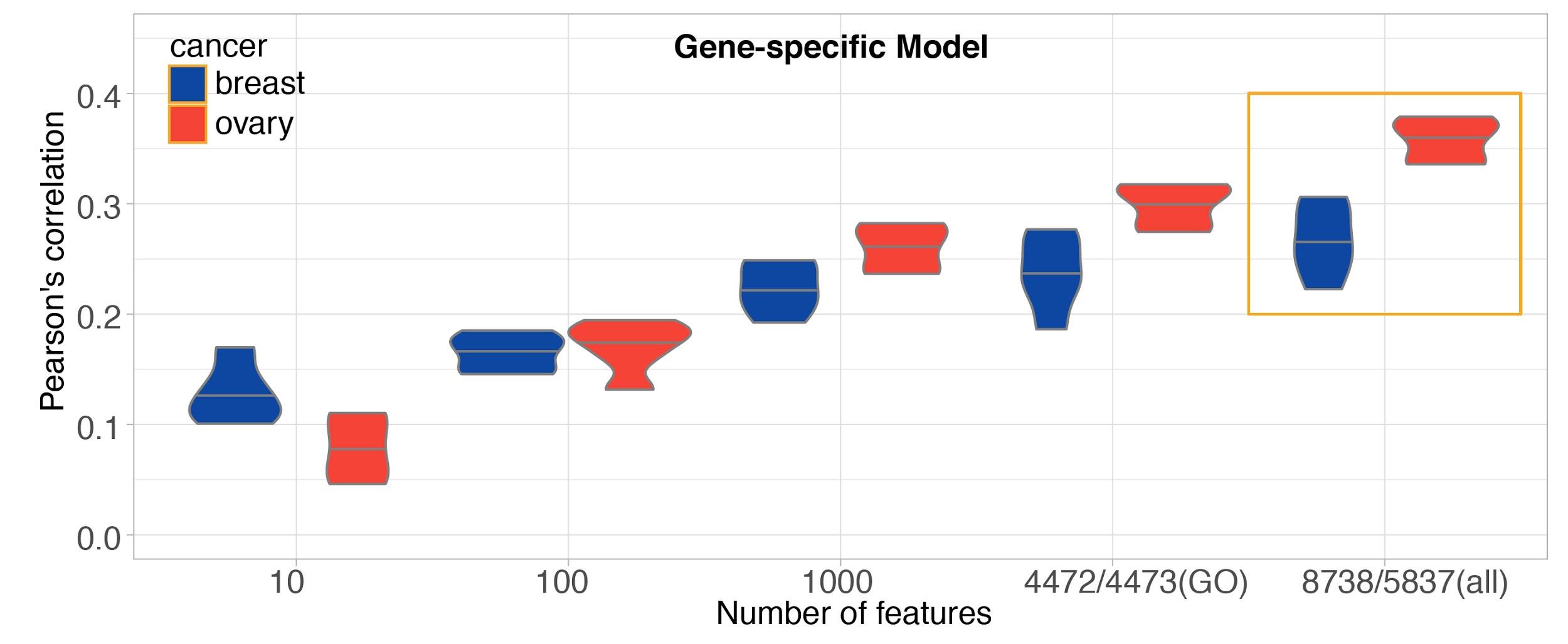


**Supplementary Figure 6. Comparison of models using different number of genes as features.**

The Pearson’s correlations of five-fold cross validation results are shown in blue (breast) and red (ovary). From left to right, the number of feature genes used in the gene-specific model increases. The first three numbers represent models using the top 10, 100, and 1,000 expressed genes as features. In addition, the gene subsets associated with GO terms (0010467: gene expression and 0010468: regulation of gene expression) are also evaluated, which contain 4,472 and 4,473 genes in breast and ovary, respectively. Our final gene-specific model uses all genes (8,738 genes in breast and 5,837 genes in ovary) as features and achieves highest correlations (the orange box).


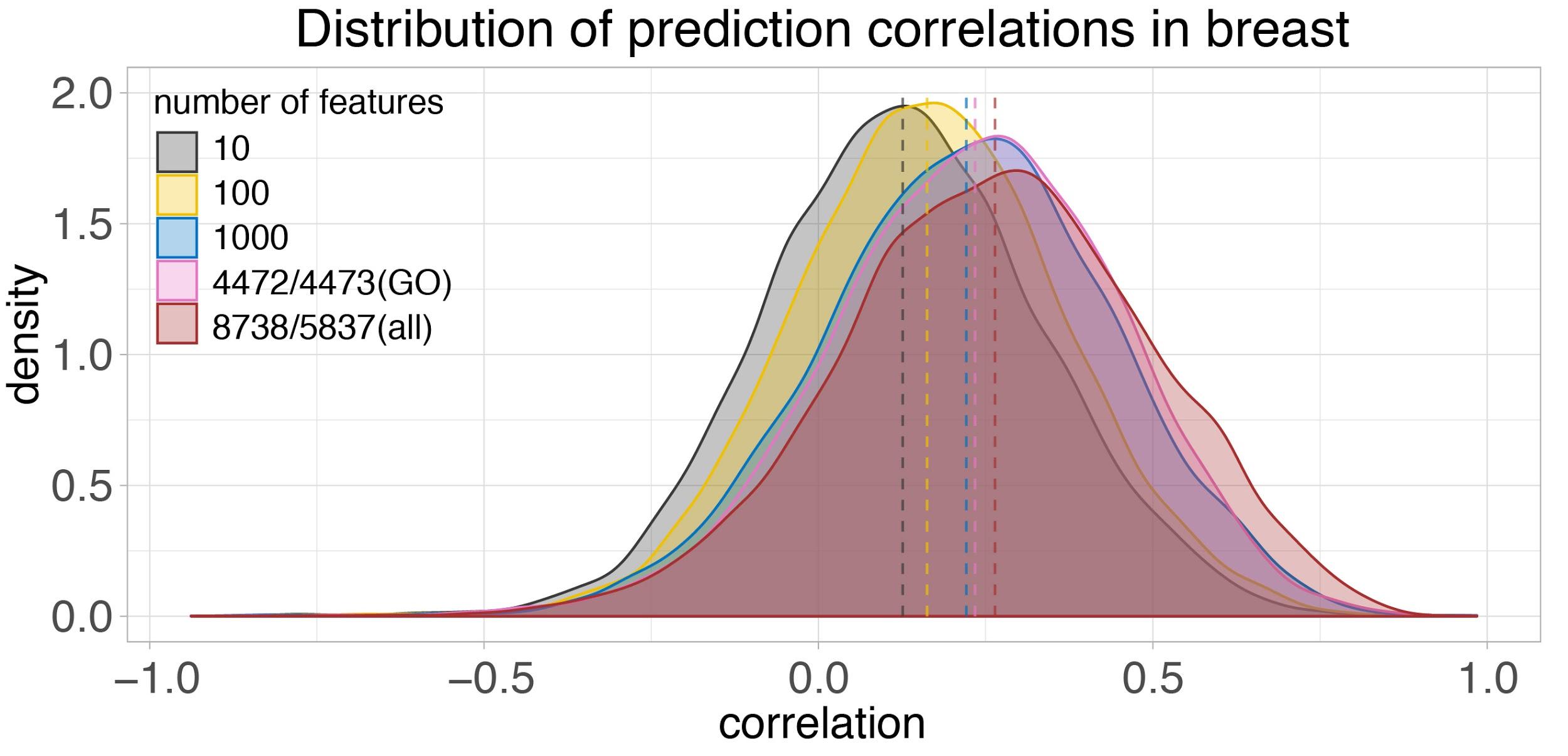


**Supplementary Figure 7. The distribution of correlations between predictions and observations across samples for all proteins in breast.**

The models using (1) the top 10 expressed genes (2) the top 100 expressed genes (3) the top 1,000 expressed genes (4) the gene subsets associated with gene expression-related GO terms (5) all genes as features are shown in five different colors. The dashed line represents the average correlation of all proteins.


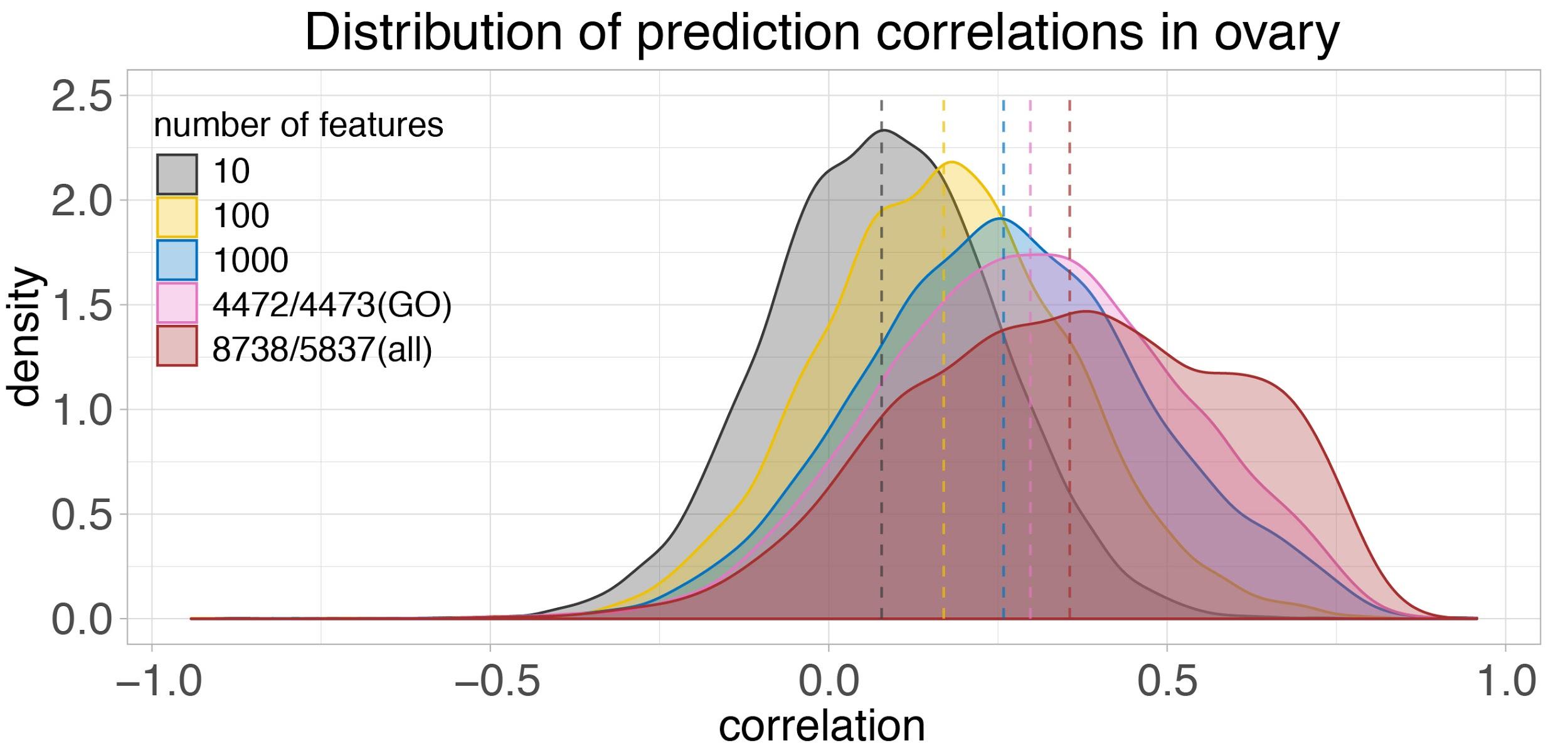


**Supplementary Figure 8. The distribution of correlations between predictions and observations across samples for all proteins in ovary.**

The models using (1) the top 10 expressed genes (2) the top 100 expressed genes (3) the top 1,000 expressed genes (4) the gene subsets associated with gene expression-related GO terms (5) all genes as features are shown in five different colors. The dashed line represents the average correlation of all proteins.


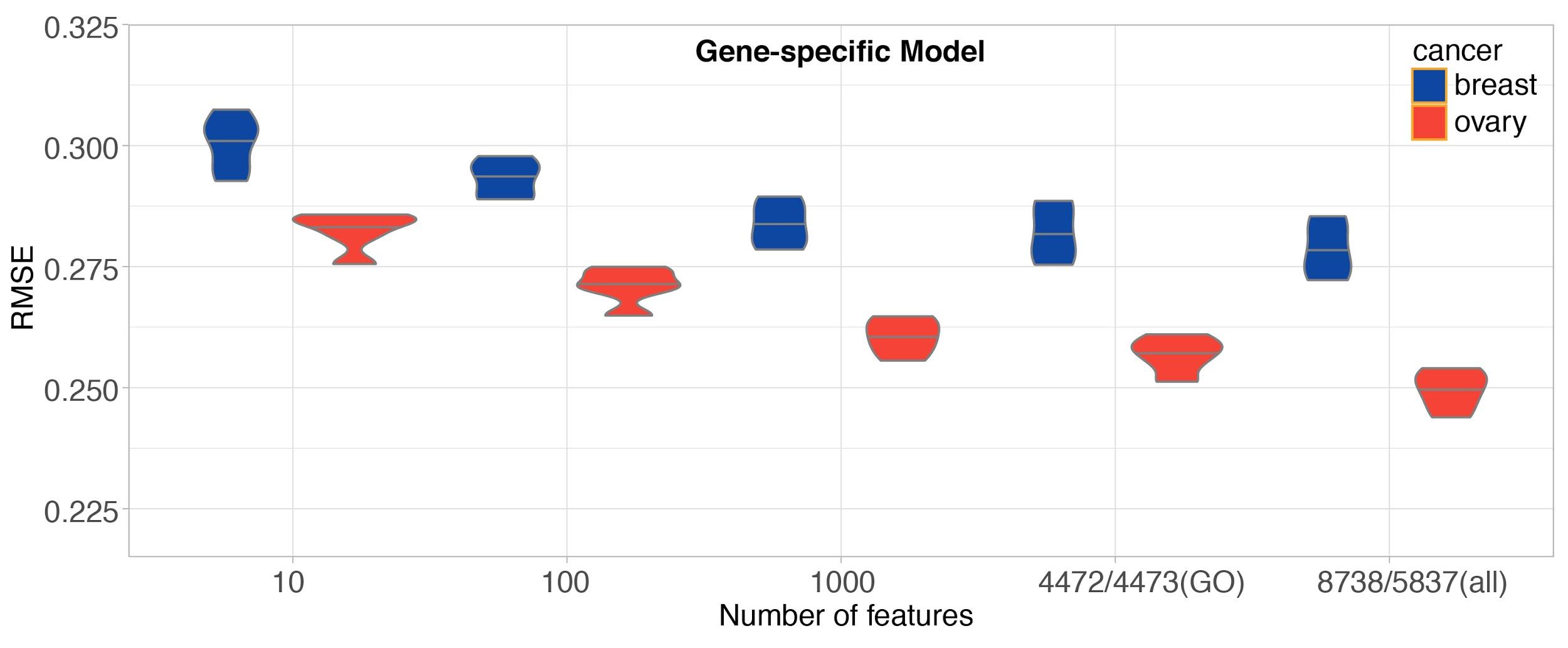


**Supplementary Figure 9. Comparison of models using different number of genes as features.**

The RMSEs of five-fold cross validation results are shown in blue (breast) and red (ovary). From left to right, the number of feature genes used in the gene-specific model increases. The first three numbers represent models using the top 10, 100, and 1,000 expressed genes as features. In addition, the gene subsets associated with GO terms (0010467: gene expression and 0010468: regulation of gene expression) are also evaluated, which contain 4,472 and 4,473 genes in breast and ovary, respectively. Our final gene-specific model uses all genes (8,738 genes in breast and 5,837 genes in ovary) as features and achieves lowest RMSEs (the orange box).


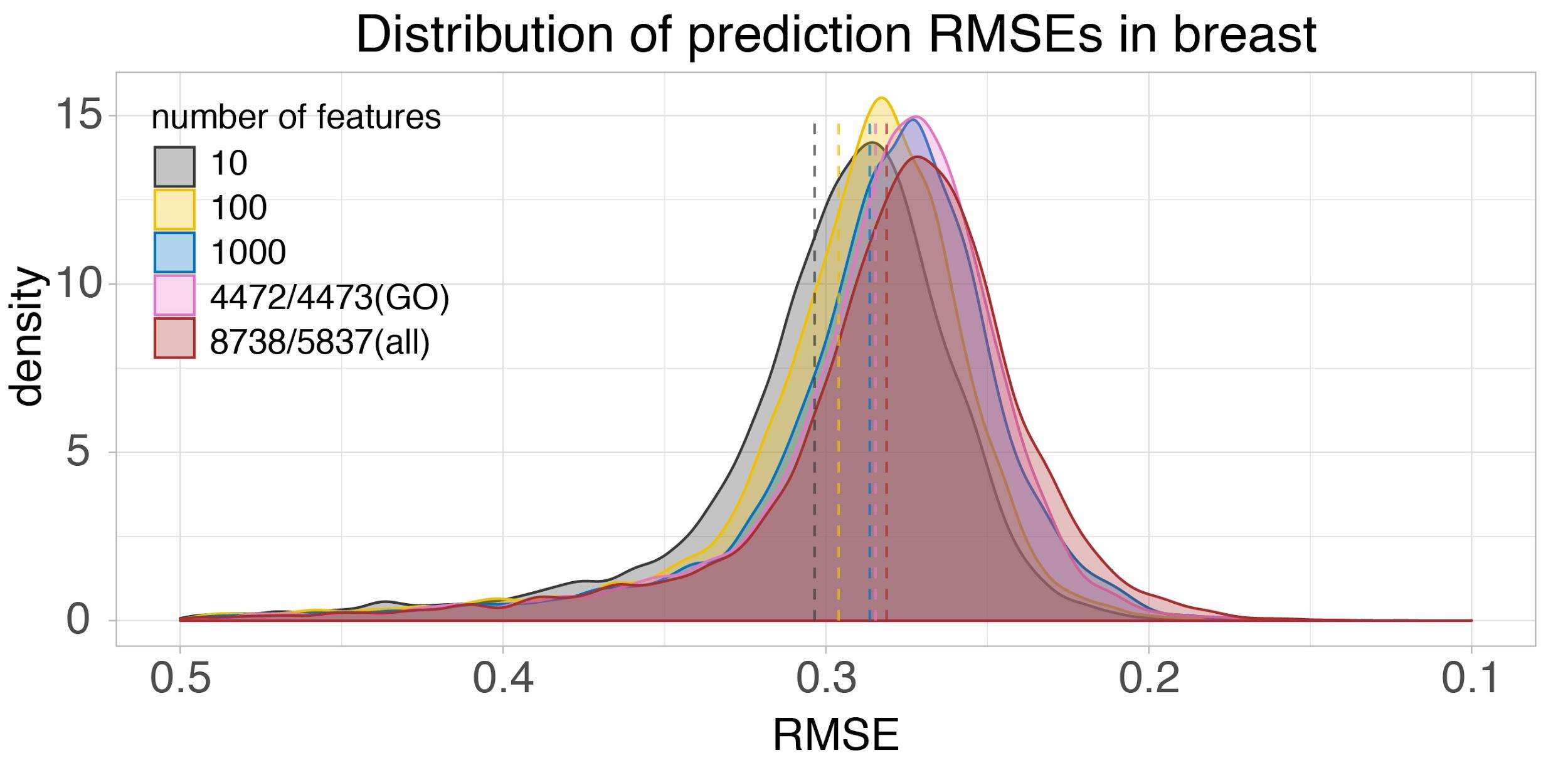


**Supplementary Figure 10. The distribution of RMSEs between predictions and observations across samples for all proteins in breast.**

The models using (1) the top 10 expressed genes (2) the top 100 expressed genes (3) the top 1,000 expressed genes (4) the gene subsets associated with gene expression-related GO terms (5) all genes as features are shown in five different colors. The dashed line represents the average RMSE of all proteins.


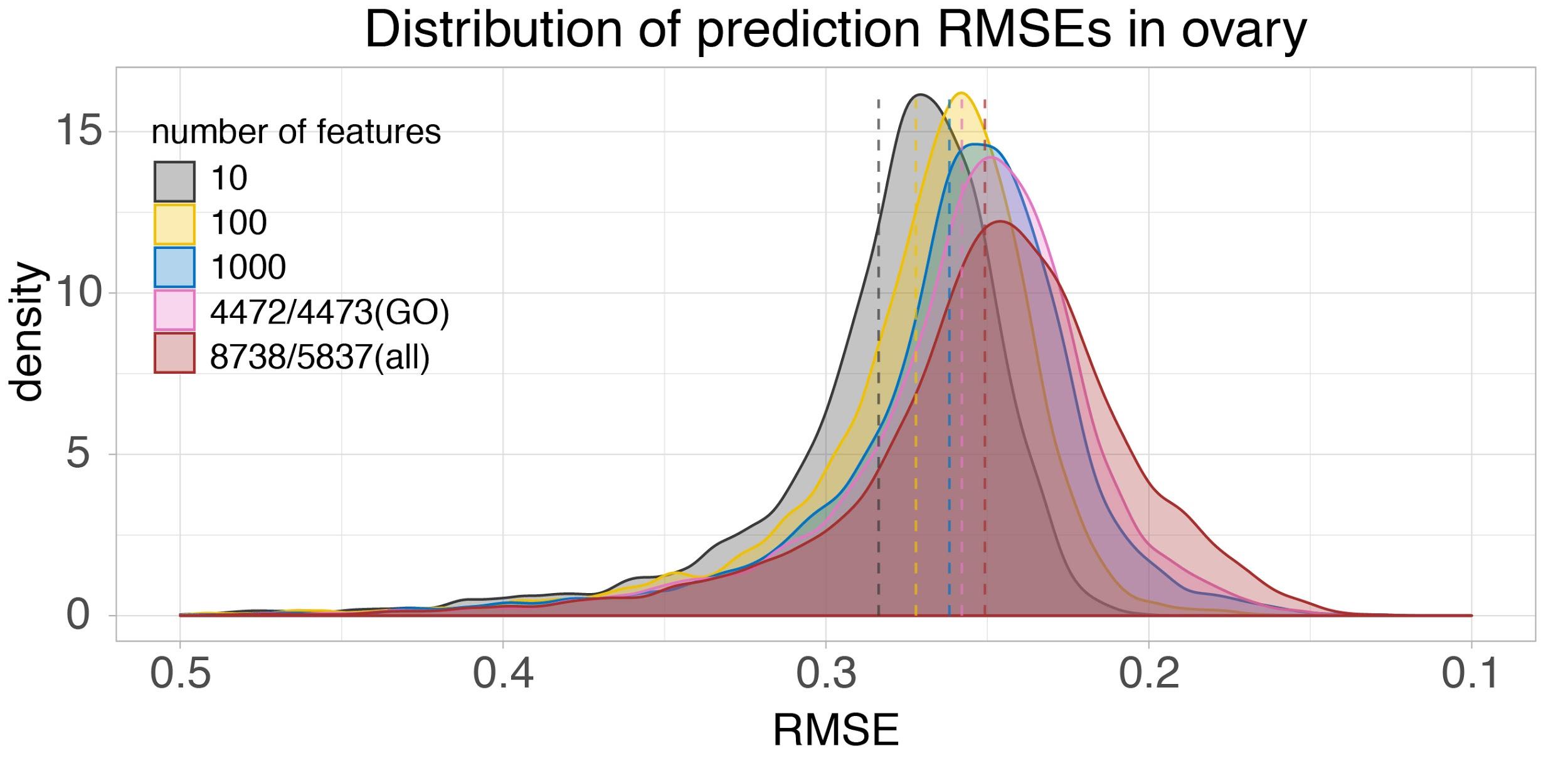


**Supplementary Figure 11. The distribution of RMSEs between predictions and observations across samples for all proteins in ovary.**

The models using (1) the top 10 expressed genes (2) the top 100 expressed genes (3) the top 1,000 expressed genes (4) the gene subsets associated with gene expression-related GO terms (5) all genes as features are shown in five different colors. The dashed line represents the average RMSE of all proteins.

**
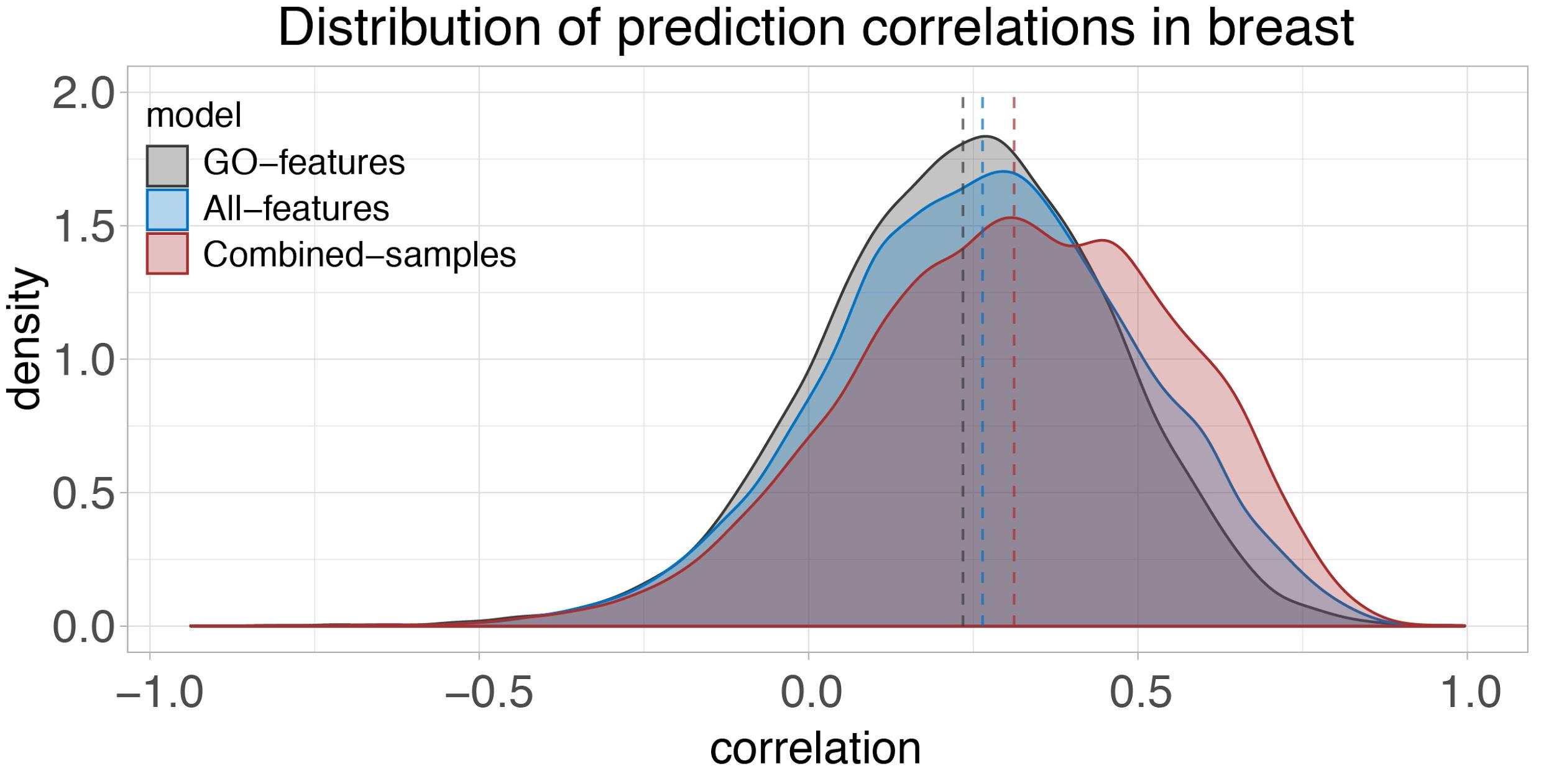
**

**Supplementary Figure 12. The distribution of correlations between predictions and observations across samples for all proteins in breast.**

The models using (1) the gene subsets associated with gene expression-related GO terms as features (2) all genes as features (3) all genes as features and combined samples from two cancer tissues are shown in different colors. The dashed line represents the average correlation of all proteins.

**
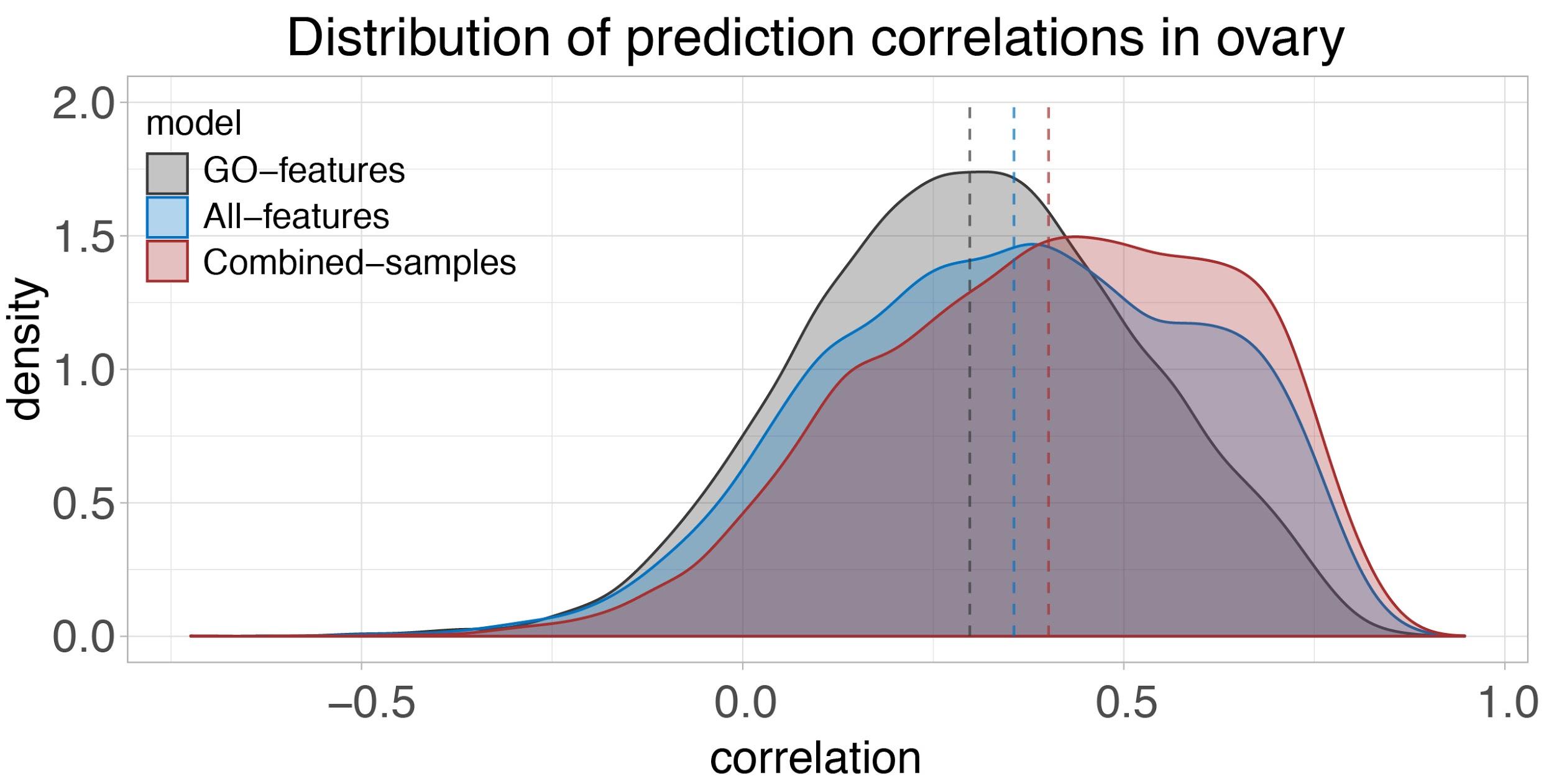
**

**Supplementary Figure 13. The distribution of correlations between predictions and observations across samples for all proteins in ovary.**

The models using (1) the gene subsets associated with gene expression-related GO terms as features (2) all genes as features (3) all genes as features and combined samples from two cancer tissues are shown in different colors. The dashed line represents the average correlation of all proteins.

**
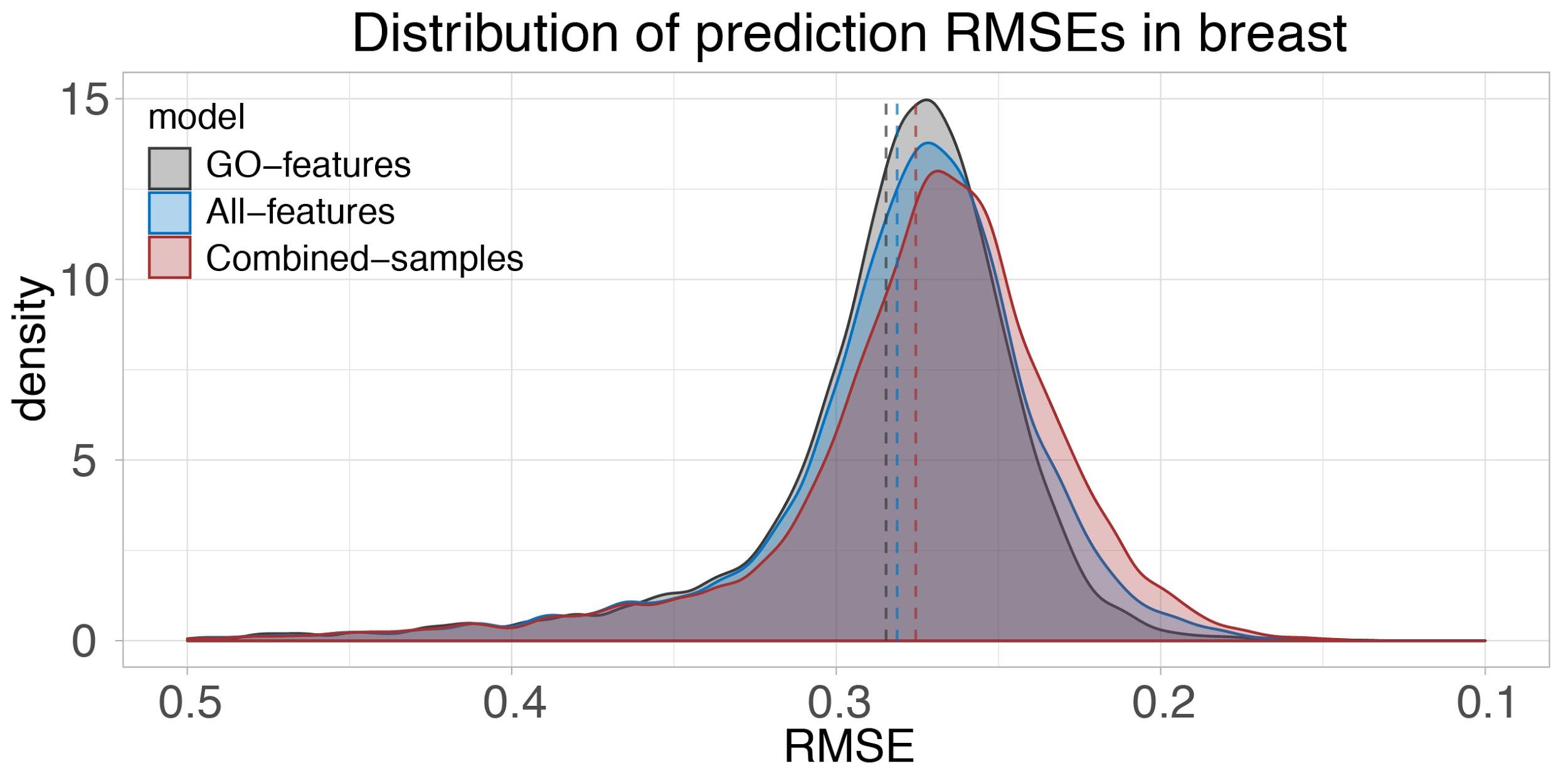
**

**Supplementary Figure 14. The distribution of RMSEs between predictions and observations across samples for all proteins in breast.**

The models using (1) the gene subsets associated with gene expression-related GO terms as features (2) all genes as features (3) all genes as features and combined samples from two cancer tissues are shown in different colors. The dashed line represents the average RMSE of all proteins.

**
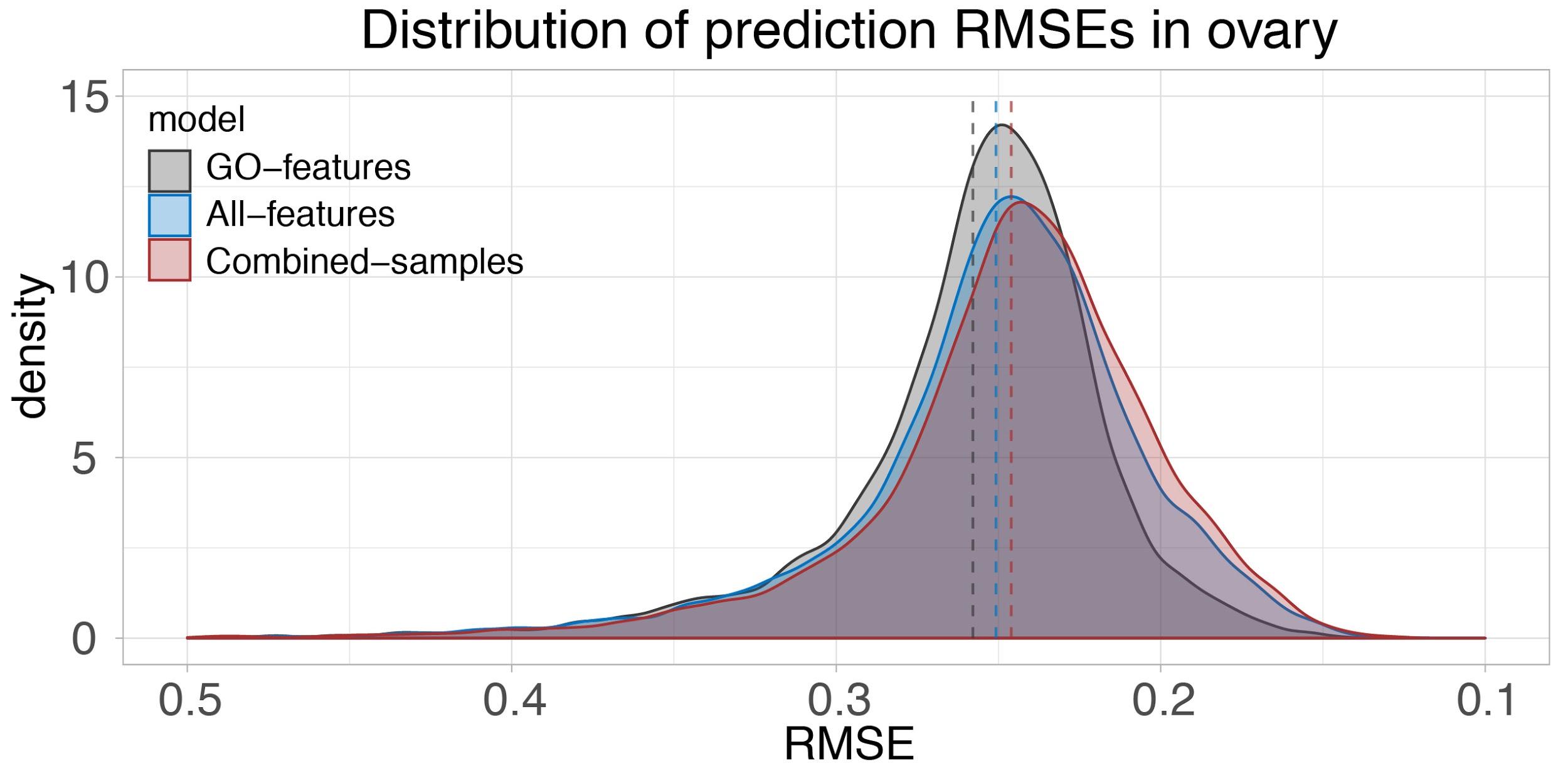
**

**Supplementary Figure 15. The distribution of RMSEs between predictions and observations across samples for all proteins in ovary.**

The models using (1) the gene subsets associated with gene expression-related GO terms as features (2) all genes as features (3) all genes as features and combined samples from two cancer tissues are shown in different colors. The dashed line represents the average RMSE of all proteins.


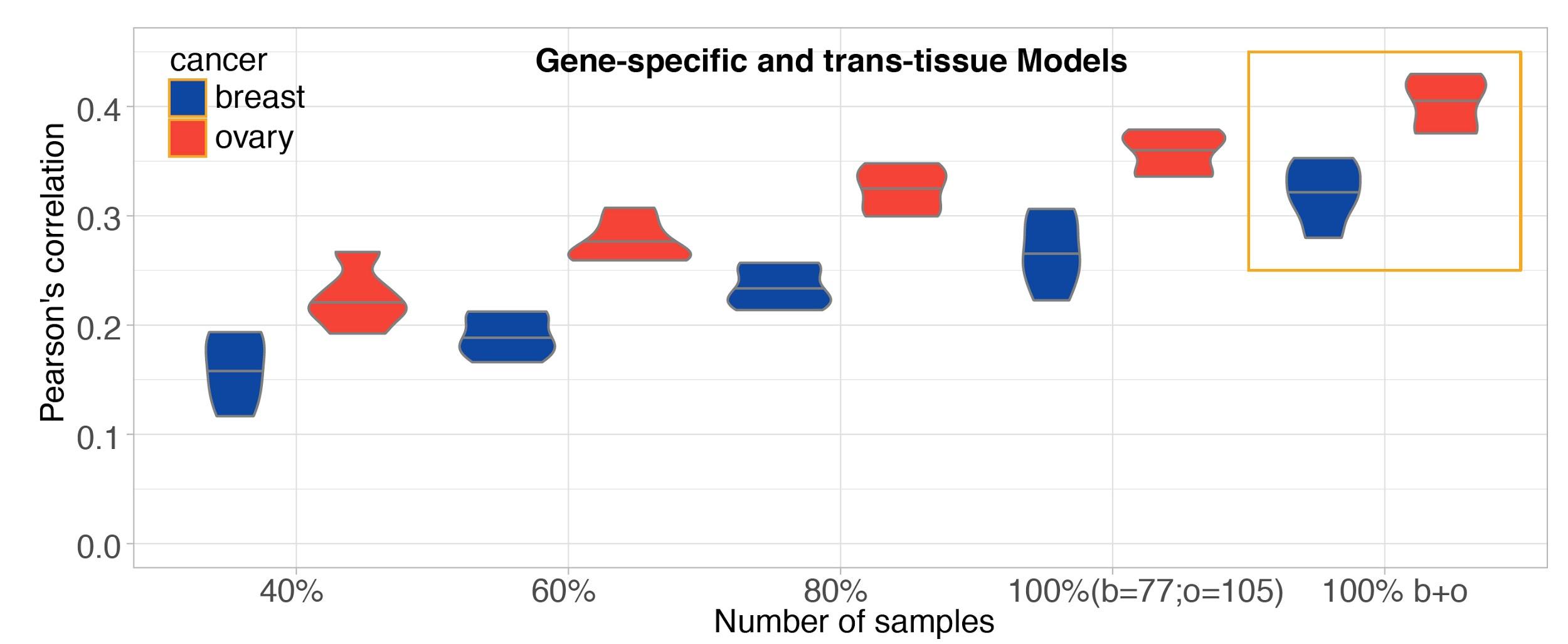


**Supplementary Figure 16. Comparison of models trained on different number of samples.**

From left to right, the Pearson’s correlations were calculated for models using (1) 40% of the training samples (2) 60% of the training samples (3) 80% of the training samples (4) 100% of the training samples (5) 100% of the combined training samples from two cancer tissues. Of note, the exact number of training samples in (4) is listed in the parentheses (b for breast and o for ovary). Our final trans-tissue model combines samples from two cancers and achieves highest correlations (the orange box).


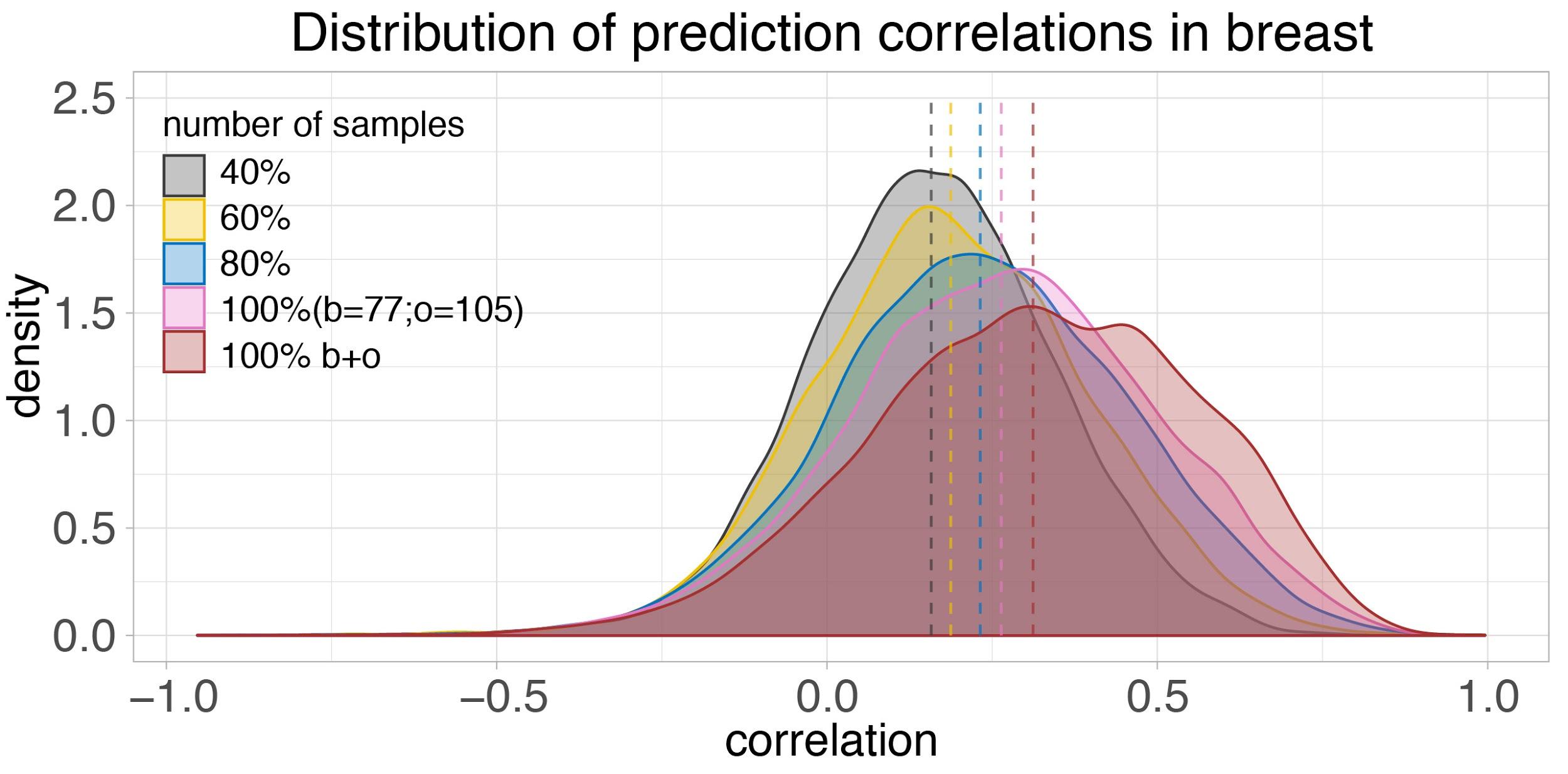


**Supplementary Figure 17. The distribution of correlations between predictions and observations across samples for all proteins in breast.**

From left to right, the models using (1) 40% of the training samples (2) 60% of the training samples (3) 80% of the training samples (4) 100% of the training samples (5) 100% of the combined training samples from two cancer tissues are shown in different colors. Of note, the exact number of training samples in (4) is listed in the parentheses (b for breast and o for ovary). The dashed line represents the average correlation of all proteins.


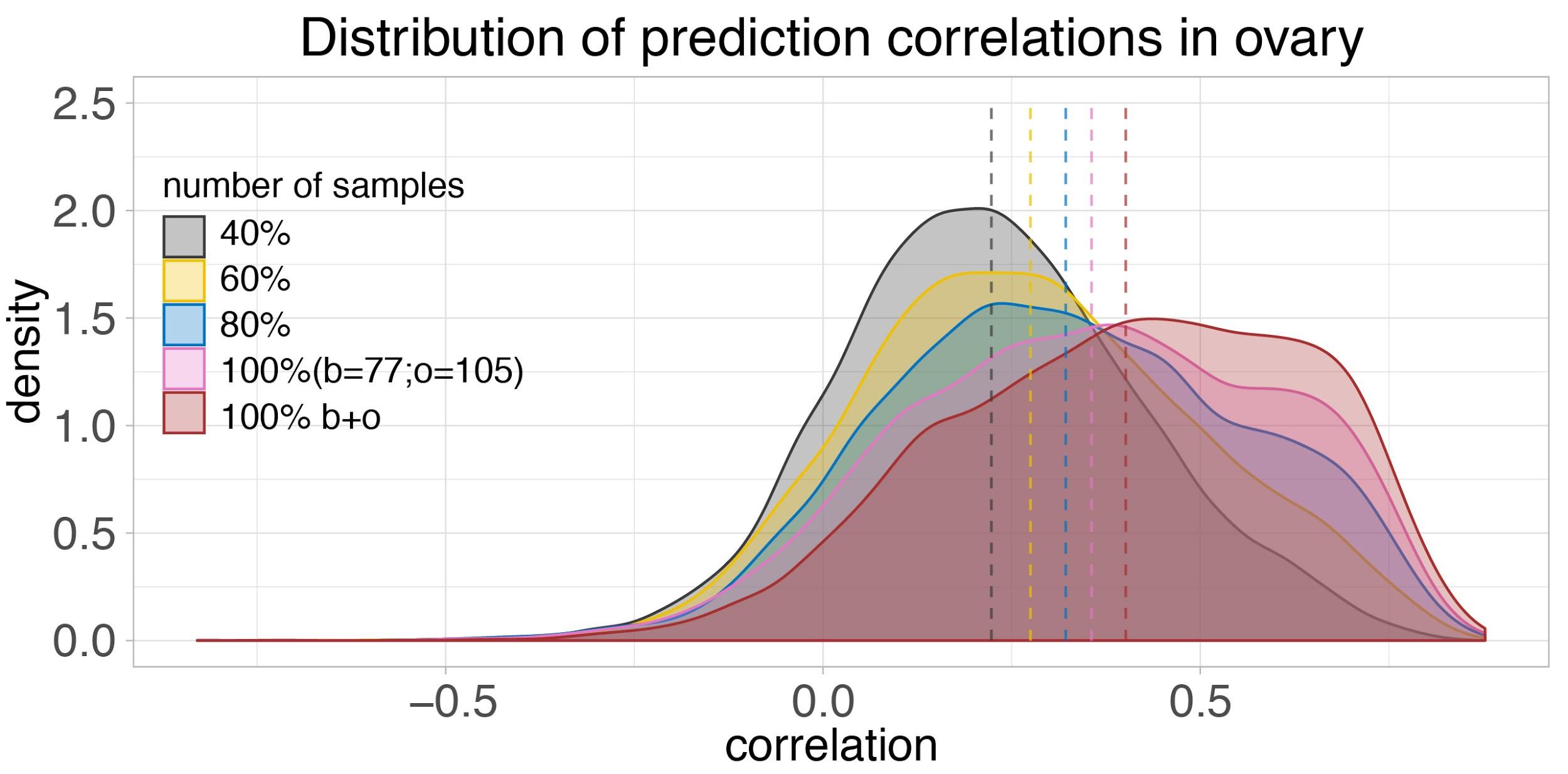


**Supplementary Figure 18. The distribution of correlations between predictions and observations across samples for all proteins in ovary.**

From left to right, the models using (1) 40% of the training samples (2) 60% of the training samples (3) 80% of the training samples (4) 100% of the training samples (5) 100% of the combined training samples from two cancer tissues are shown in different colors. Of note, the exact number of training samples in (4) is listed in the parentheses (b for breast and o for ovary). The dashed line represents the average correlation of all proteins.


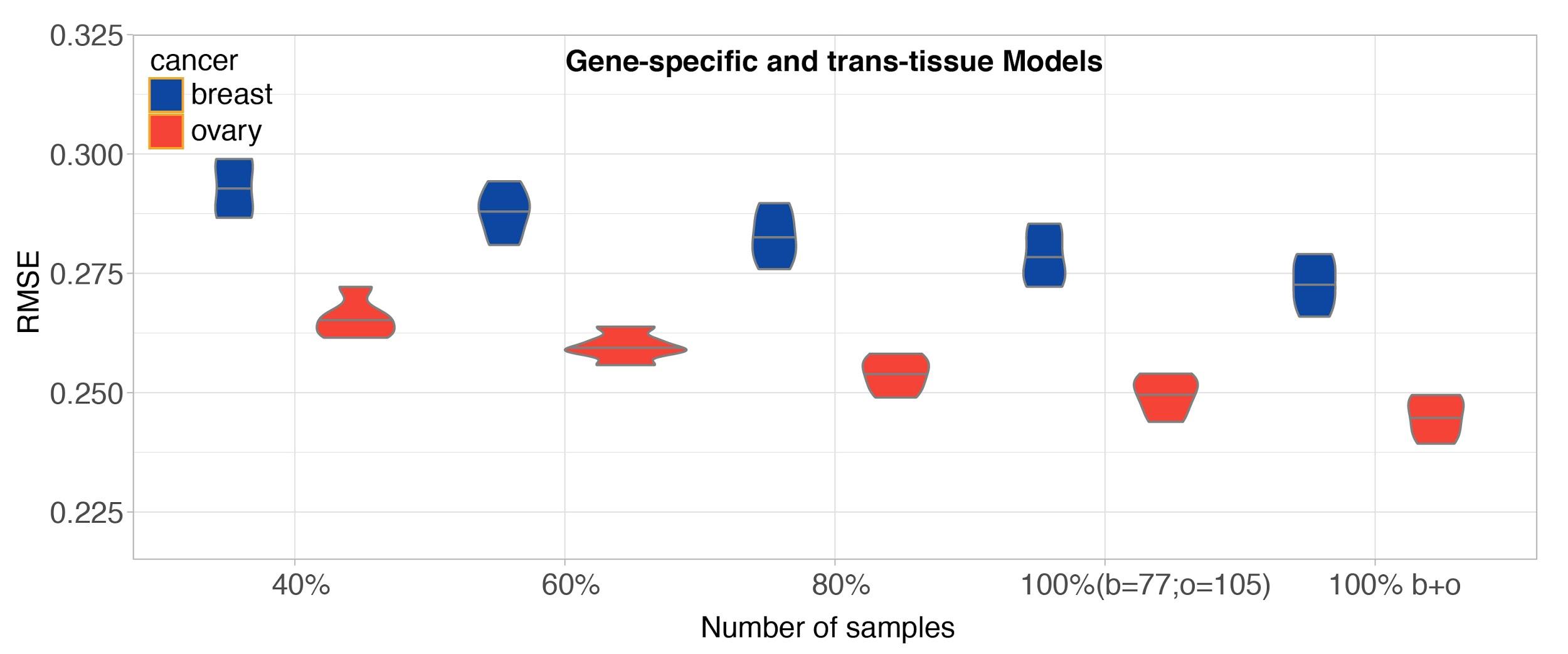


**Supplementary Figure 19. Comparison of models trained on different number of samples.**

From left to right, the RMSEs were calculated for models using (1) 40% of the training samples (2) 60% of the training samples (3) 80% of the training samples (4) 100% of the training samples (5) 100% of the combined training samples from two cancer tissues. Of note, the exact number of training samples in (4) is listed in the parentheses (b for breast and o for ovary). Our final trans-tissue model combines samples from two cancers and achieves lowest RMSEs.


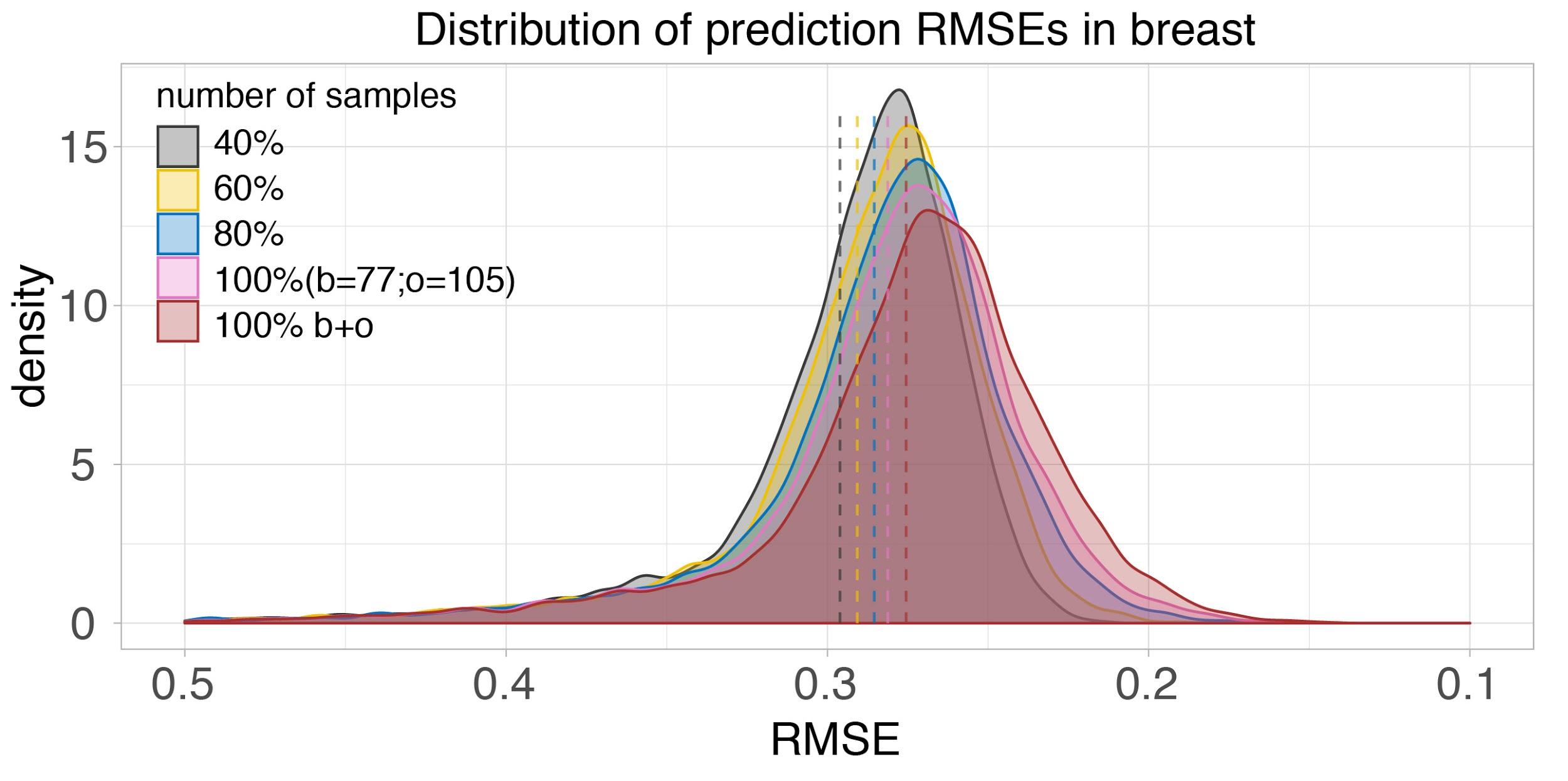


**Supplementary Figure 20. The distribution of RMSEs between predictions and observations across samples for all proteins in breast.**

From left to right, the models using (1) 40% of the training samples (2) 60% of the training samples (3) 80% of the training samples (4) 100% of the training samples (5) 100% of the combined training samples from two cancer tissues are shown in different colors. Of note, the exact number of training samples in (4) is listed in the parentheses (b for breast and o for ovary). The dashed line represents the average RMSE of all proteins.

**
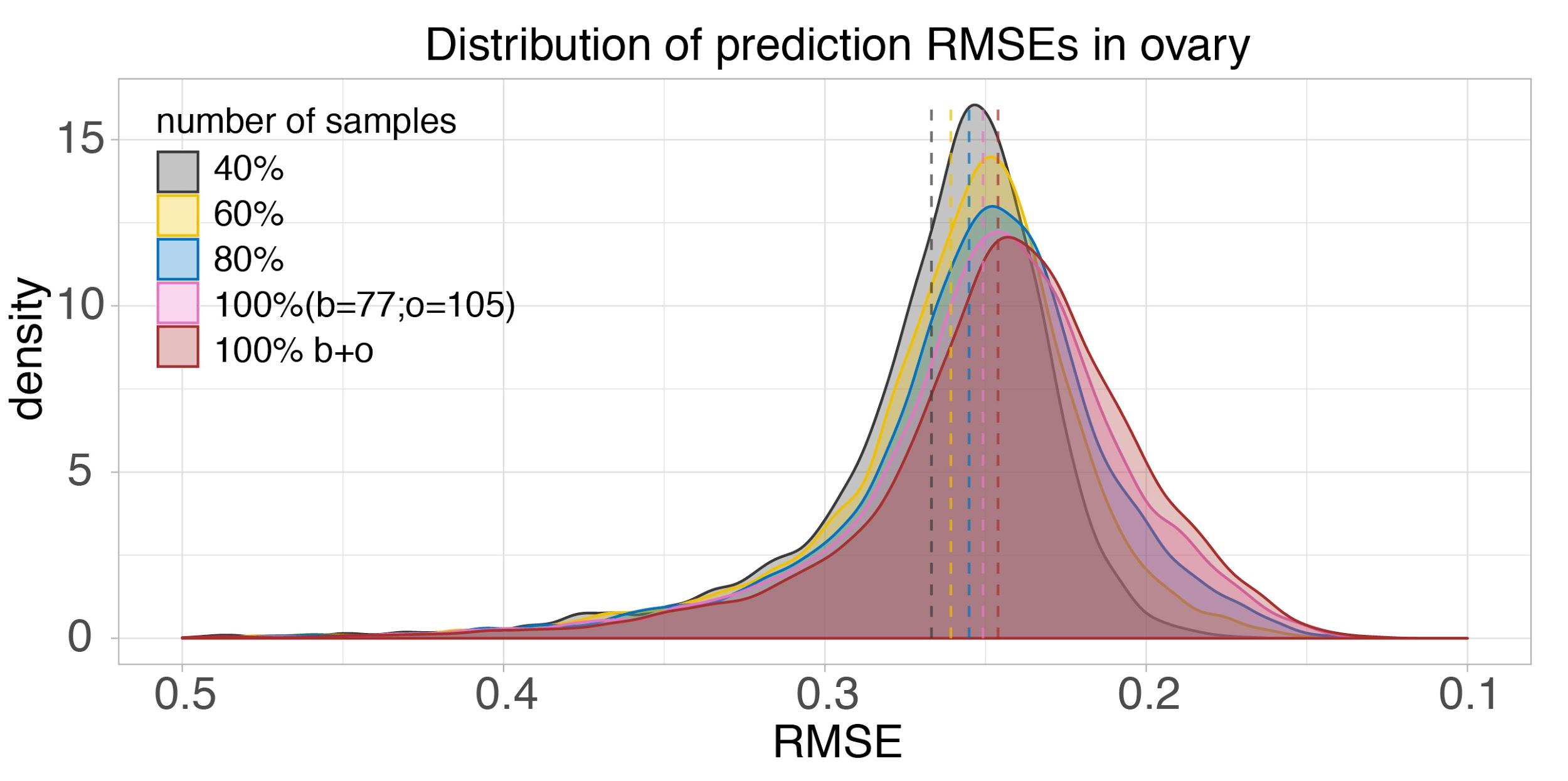
**

**Supplementary Figure 21. The distribution of RMSEs between predictions and observations across samples for all proteins in ovary.**

From left to right, the models using (1) 40% of the training samples (2) 60% of the training samples (3) 80% of the training samples (4) 100% of the training samples (5) 100% of the combined training samples from two cancer tissues are shown in different colors. Of note, the exact number of training samples in (4) is listed in the parentheses (b for breast and o for ovary). The dashed line represents the average RMSE of all proteins.

**
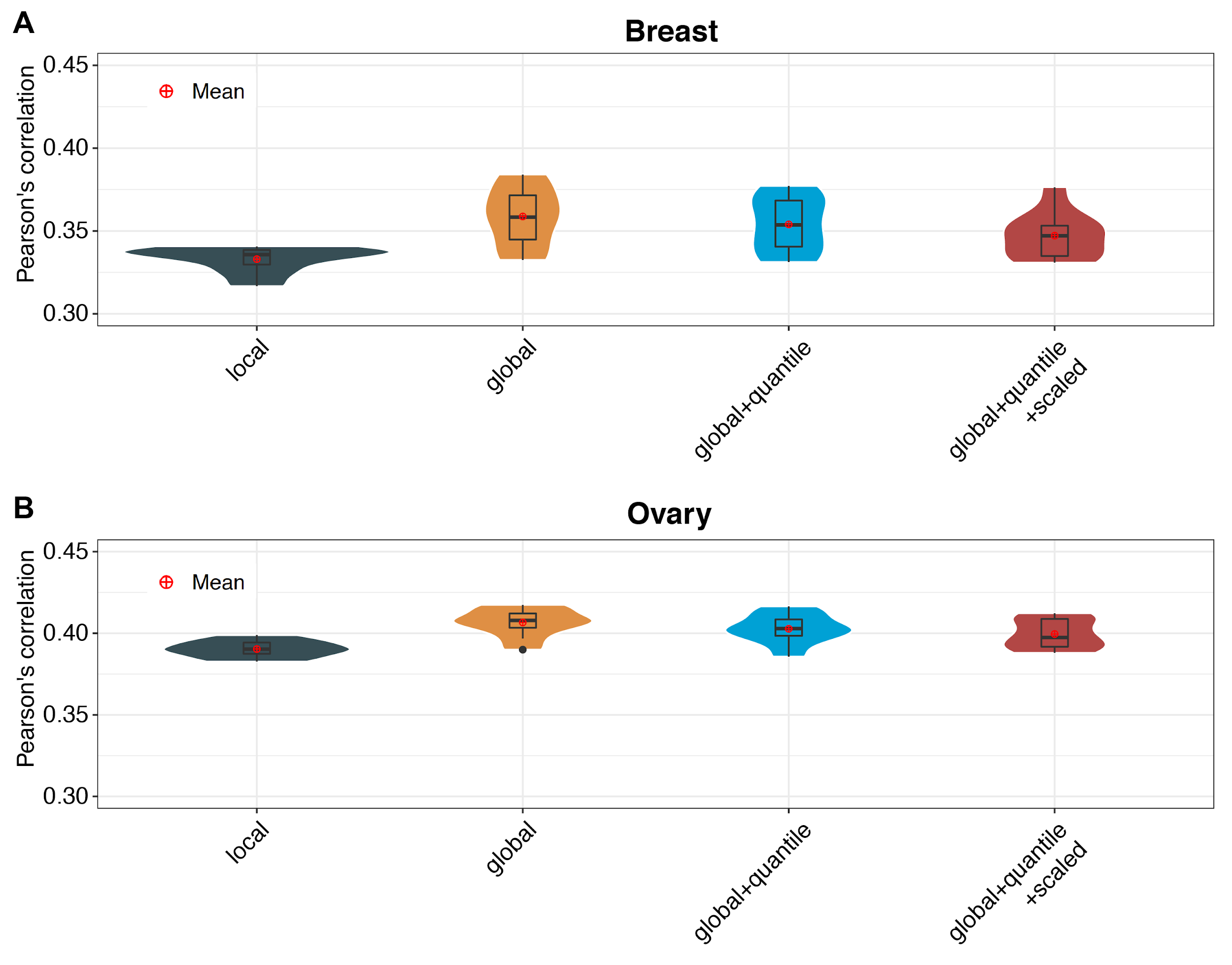
**

**Supplementary Figure 22. The effects of different training scenarios and normalization strategies.**

**A-B**. The random forest model was trained on the two features, RNA and CNV values of a gene in (**A**) breast and (**B**) ovary. Two training scenarios were applied: 1. in the “local” model, only the samples of the same gene were used to train and 2. in the “global” model, the samples of all genes were used to train. To reduce the potential batch effects across individuals, in the “global+quantile” model, we quantile mapped the RNA or CNV profile of each individual to the corresponding reference profile, which was the average expression level across all individuals. We further tested the effects of adjusting the overall expression profile of an individual by multiplying an individual-specific ratio.


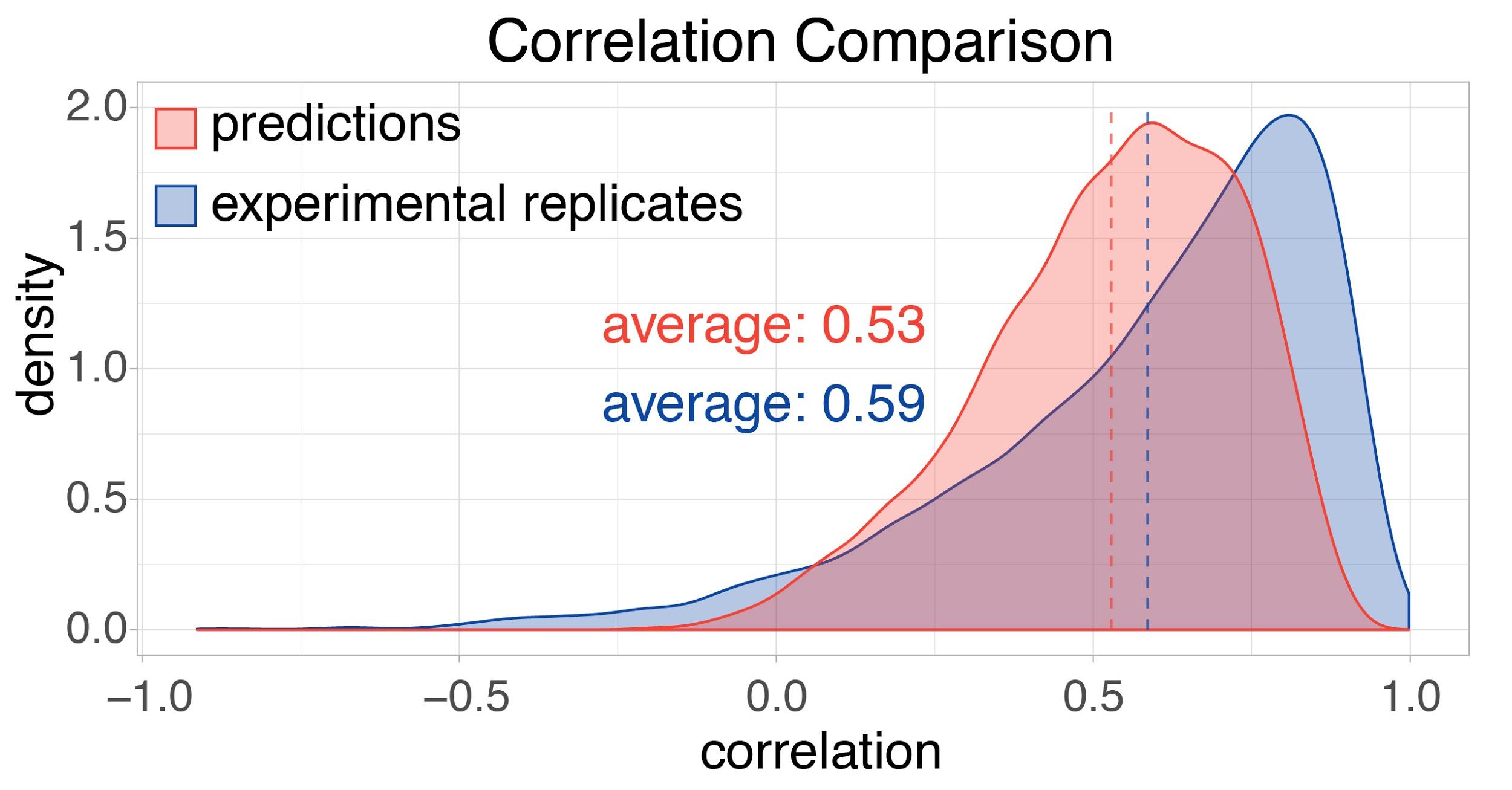


**Supplementary Figure 23. The comparison of predictions by our method and experimental replicates.**

We calculated the Pearson’s correlations across 32 overlapping ovarian cancer samples measured at both JHU and PNNL for all proteins (blue). Meanwhile, the prediction correlation of our method on the held-out testing dataset during the NCI-CPTAC DREAM challenge were shown in red. The dashed line represents the average correlation.


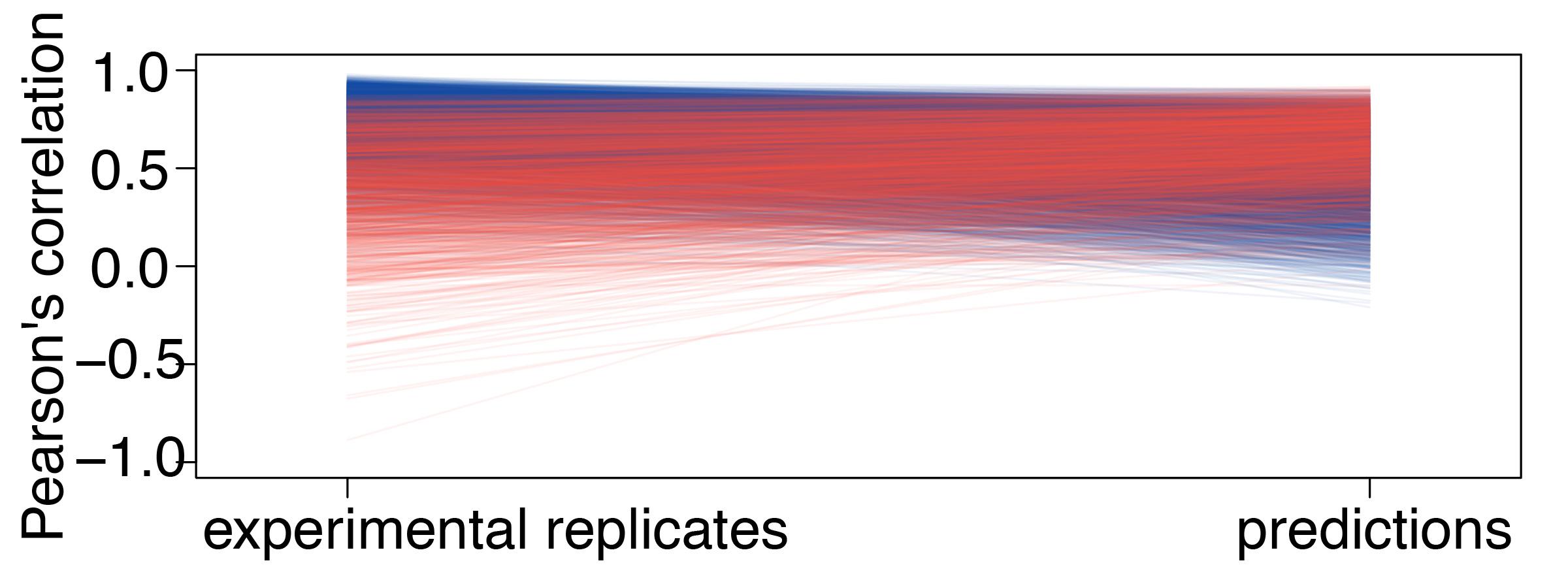


**Supplementary Figure 24. The pair-wise comparison of predictions by our method and experimental replicates.**

We calculated (1) the Pearson’s correlations across 32 overlapping ovarian cancer samples measured at both JHU and PNNL for all proteins. (2) the prediction correlations of our method on the held-out testing dataset during the NCI-CPTAC DREAM challenge. Each protein is shown as a line connecting the correlation values calculated from (1) and (2). For the 5,218 proteins under consideration, 3,775 (72.3%) proteins have higher correlations between experimental replicates than our predictions (blue lines). The other 1,443 (27.6%) proteins have lower correlations between experimental replicates than our predictions (red lines), due to the intrinsic noises and fluctuations in the experimental data.


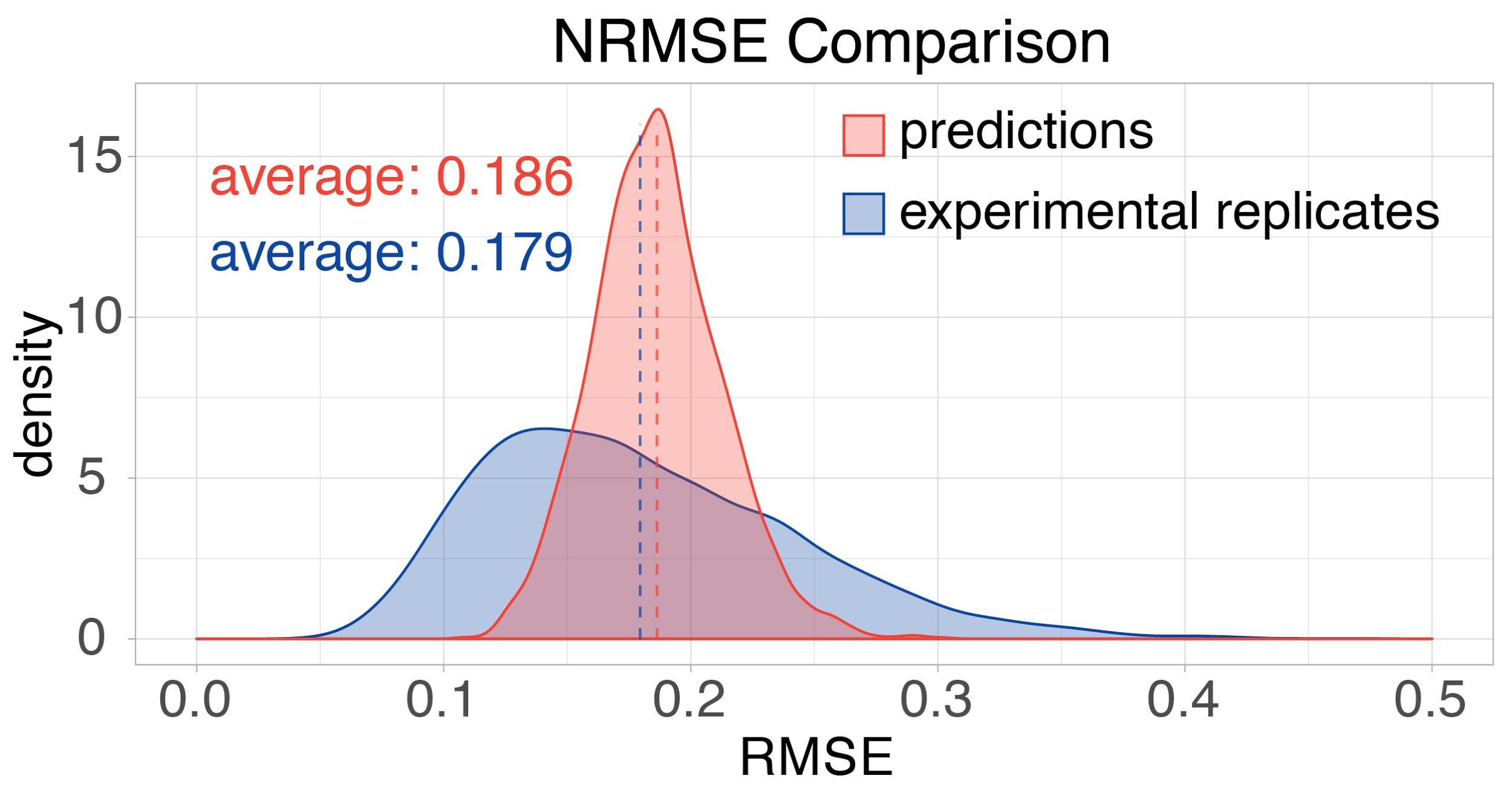


**Supplementary Figure 25. The comparison of predictions by our method and experimental replicates.**

We calculated the RMSEs across 32 overlapping ovarian cancer samples measured at both JHU and PNNL for all proteins (blue). Meanwhile, the prediction RMSEs of our method on the held-out testing dataset during the NCI-CPTAC DREAM challenge were shown in red. The two dashed line represents the average RMSE.


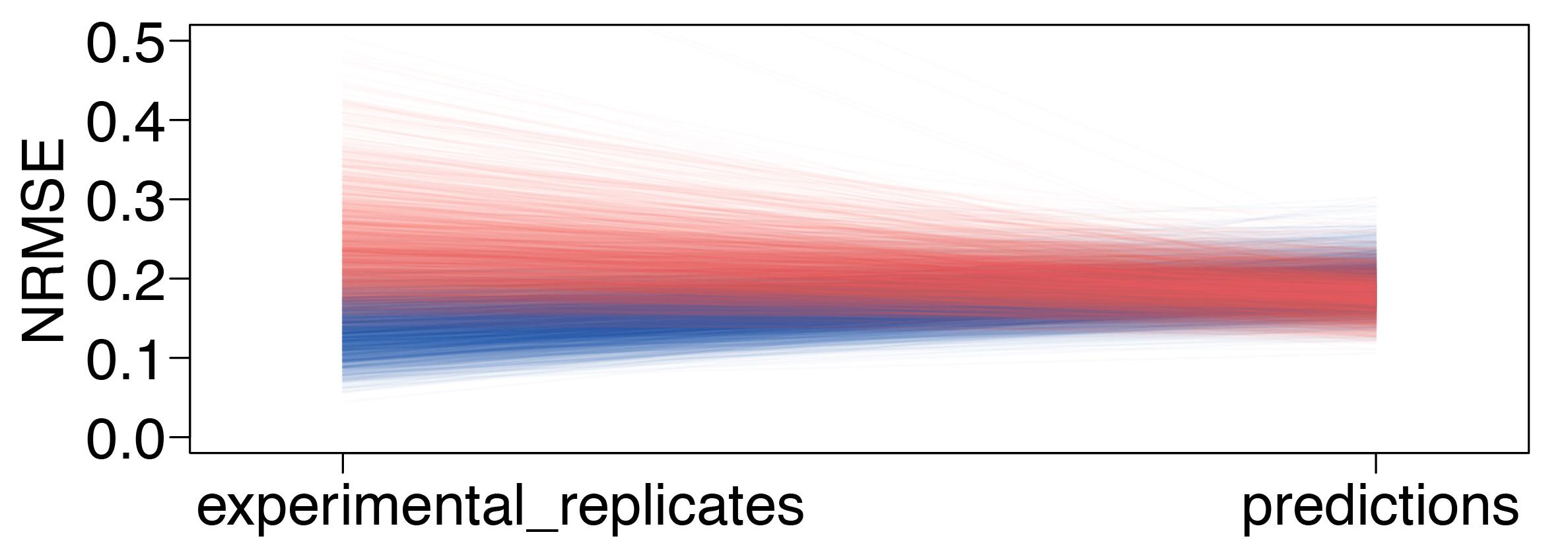


**Supplementary Figure 26. The pair-wise comparison of predictions by our method and experimental replicates.**

We calculated (1) the RMSEs across 32 overlapping ovarian cancer samples measured at both JHU and PNNL for all proteins. (2) the prediction RMSEs of our method on the held-out testing dataset during the NCI-CPTAC DREAM challenge. Each protein is shown as a line connecting the RMSE values calculated from (1) and (2). For the 5,218 proteins under consideration, 2,930 (56.1%) proteins have lower RMSEs between experimental replicates than our predictions (blue lines). The other 2,288 (43.8%) proteins have higher RMSEs between experimental replicates than our predictions (red lines), due to the intrinsic noises and fluctuations in the experimental data.

**
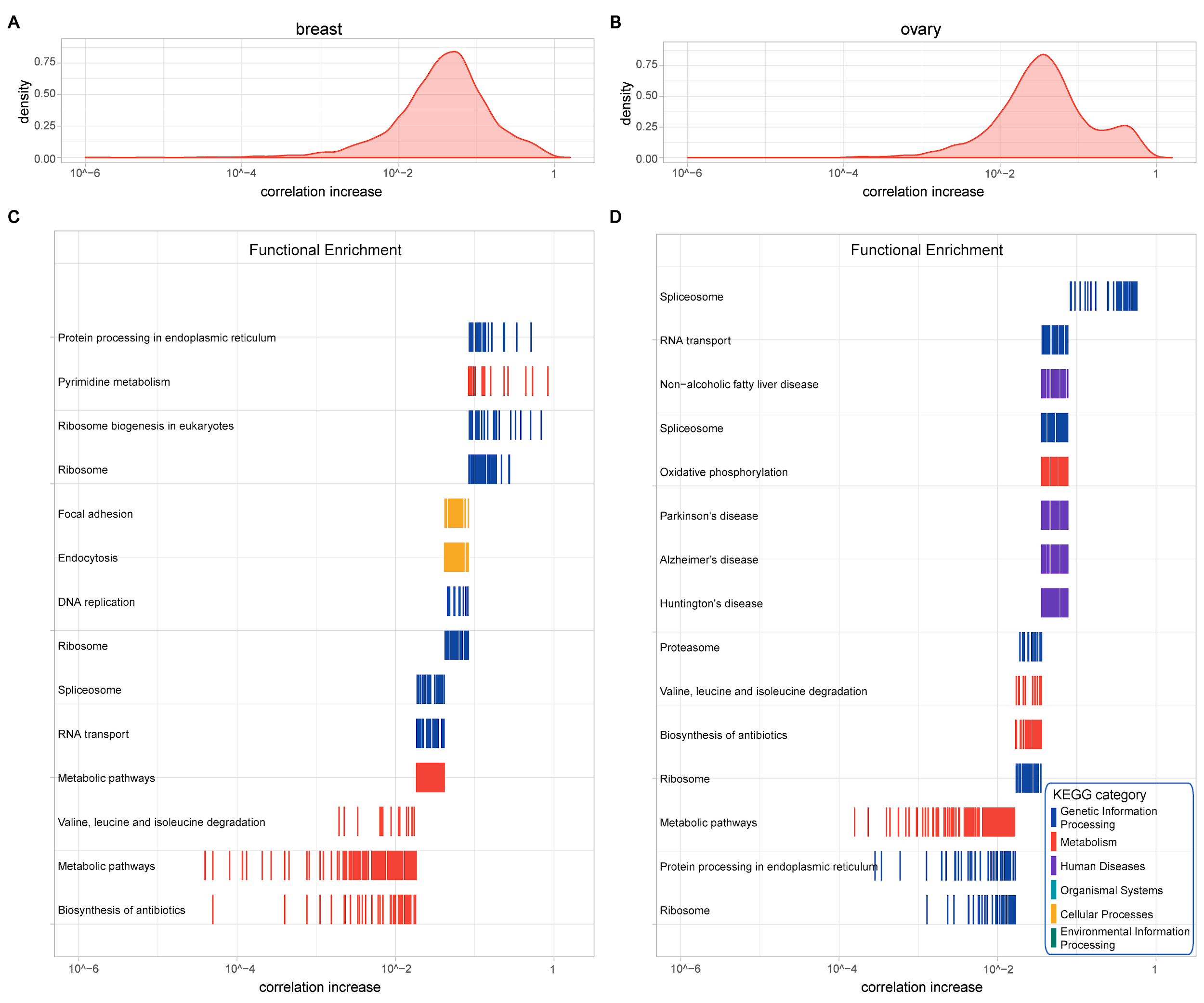
**

**Supplementary Figure 27. The functional enrichment analysis of gene sets with different correlation increases.**

The overall distribution of the Pearson’s correlation increases using our method, compared with the baseline mRNA-protein correlation in **A**. breast and **B**. ovarian cancers. **C-D**. Functional enrichment analysis was performed on gene subsets based on the improvement.

**Supplementary Table 1. The five-fold Pearson’s correlations of the generic, gene-specific and trans-tissue models.**

**Supplementary Table 2. The five-fold correlations of models using different numbers of features.**

**Supplementary Table 3. The weighting ratios to stack the generic, gene-specific and trans-tissue models and the corresponding prediction correlations in breast.**

For example, 1_3_7 represents the stacking ratios is 1:3:7 and the five numbers below it are the 5-fold cross validation results.

**Supplementary Table 4. The weighting ratios to stack the generic, gene-specific and trans-tissue models and the corresponding prediction correlations in ovary.**

**Supplementary Table 5. The five-fold correlations of models using different numbers of samples.**

**Supplementary Table 6. The correlations and RMSEs across 32 overlapping ovarian cancer samples measured at both JHU and PNNL.**

The first column is the gene name. The 2nd and 3rd columns are the correlation and RMSE, respectively. Of note, if a gene has a “NA” value, it means this gene does not have enough number of overlapping observations in the two cohorts. For correlation calculation, a minimum number of 5 observations is required. For RMSE calculation, a minimum of 70% observations (32*0.7) is required.

**Supplementary Table 7. The correlations and RMSEs of our predictions on the held-out testing dataset of 82 ovarian cancer samples during the NCI-CPTAC DREAM challenge.**

The first column is the gene name. The 2nd and 3rd columns are the correlation and RMSE, respectively.

**Supplementary Table 8. The feature importance of all genes in breast.**

**Supplementary Table 9. The feature importance of all genes in ovary.**

**Supplementary Table 10. The list of driver genes and their clusters in the breast functional network.**

**Supplementary Table 11. The list of driver genes and their clusters in the ovary functional network.**
